# Revising Model Reactions in Plasmonic Chemistry: From Nitrothiophenol Coupling to Alkoxyamine Homolysis

**DOI:** 10.1021/acscatal.5c01129

**Published:** 2025-06-13

**Authors:** Alina Gorbunova, Daria E. Votkina, Oleg Semyonov, Dmitry Kogolev, Jean-Patrick Joly, Sylvain R. A. Marque, Junais Habeeb Mokkah, Soniya Gahlawat, Markus Valtiner, Odile Chevalier, Pavel S. Postnikov, Olga Guselnikova

**Affiliations:** † Research School of Chemistry and Applied Biomedical Sciences, 65078 Tomsk Polytechnic University, Tomsk 634050, Russian Federation; ‡ Institute of Applied Physics, Vienna University of Technology, Vienna 1040, Austria; § Aix-Marseille University, CNRS, UMR 7273, ICR case 551, Avenue Escadrille Normandie-Niemen, Marseille 13397 Cedex 20, France; ∥ College of Integrative Studies, 659537 Abdullah Al Salem University (AASU), Block 3, Khaldiya 72303, Kuwait; ⊥ 128791 Aix-Marseille University, Avenue Escadrille Normandie-Niemen, Marseille 13397 Cedex 20, France; # Department of Solid-State Engineering, Institute of Chemical Technology, Prague 16628, Czech Republic

**Keywords:** alkoxyamine, azo coupling, photocatalysis, plasmon catalysis, surface plasmon resonance

## Abstract

The progress in plasmonic chemistry requires research on energy transfer, mechanisms, and materials discovery. In this pursuit, there are >3000 papers applying the azo coupling of 4-nitrothiophenol (PNTP) as a model reaction. Here, we challenge the status of this reaction as a model due to experimental evidence of thiol desorption during plasmon excitation using laser irradiation monitored by X-ray photoelectron spectroscopy (XPS) as an analytic technique. The azo coupling was performed on commonly used Au nanoparticles (NPs) coated with PNTP and confirmed by Raman spectroscopy and XPS. Changes in the N 1s and S 2p spectral regions indicated the cleavage of the Au–S bond, accompanied by thiol oxidation. Based on XPS data, we hypothesized a chemical pathway and a kinetic model that surpasses previously used simple models in complexity, making it challenging to draw reliable conclusions. The dissociation of the Au–S bond is triggered by plasmonic heating, supported by experimentally and theoretically determined local temperatures exceeding the thiol desorption temperature. The azo coupling reaction does not fit within the requirements of the model one, which should be simple and proceed with structurally evidenced products. As one of the alternative reactions, we suggest alkoxyamine homolysis tracked by electron paramagnetic resonance spectroscopy because of known products and the simple kinetic model. Applications of suitable model reactions accelerate discoveries in plasmon catalysis.

## Graphical abstract

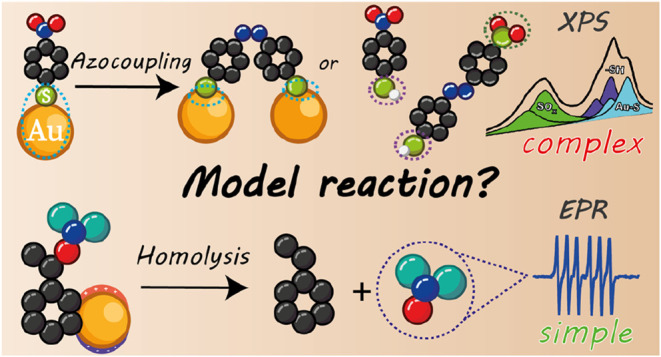

## Introduction

The phenomenon of plasmon excitation on the surface of metal nanostructures (NSs) becomes a starting point for the field of plasmon catalysis, which seeks to maximize the conversion of light to a wide range of chemical reactions with desired selectivity. Originating from plasmon-induced charge separation on Au–Semiconductor interfaces for water splitting,^1–3^ NSs have become an appealing catalyst for many reactions. To improve the plasmonic performance, mechanistic studies considering the effects of various factors, such as the wavelength, environment, irradiation mode, morphology, size, and composition of NSs, have been performed by many groups,^4–7^ including ours.^8–11^

To gather the data needed to improve the catalytic performance, the model reactions were widely applied. Historically, one of the first model reactions for studying mechanisms of plasmon catalysis was the azo coupling between two molecules of *p*-amino/nitrothiophenol (PATP/PNTP) attached to the Ag, Au, and Cu nanoparticles (NPs), leading to the formation of 4,4′-dimercaptoazobenzene (DMAB).^12–14^ Such a reaction became a base for the various kinetic studies for mechanistic insights shedding the light on the effects of the pH,^15^ environment,^6^ laser intensity,^16^ laser wavelength,^17,18^ temperature,^19^ and nature of plasmon-active substrates^20^ and NSs.^21–23^ All of these reports have been based on the evaluation of plasmonic reaction rates under varied parameters, providing the conclusion about the effect of these parameters.

To prepare the substrate for azo coupling, PATP/PNTP is assembled on a plasmonic metal surface via chemisorption with the formation of a metal–thiol bond (209.3 kJ/mol).^24,25^ Despite the benefits of thiolate self-assembled monolayers (SAMs) making them natural workhorse molecular linkers in various fields of nanoscience,^25,26^ there are issues with treating this reaction as a model. The stability of Au–S bonds under various conditions, such as plasmon excitation with energy release, is questionable. According to previous reports,^24^ the main challenge of thiolate SAMs on metals is chemical and thermal stability. These SAMs can be cleaved under the elevated temperatures,^27–29^ pH,^30^ and UV irradiation.^31^ Despite some alarming reports showing thiol desorption from noble metal NSs under plasmon excitation using femtosecond irradiation^32,33^ and continuous irradiation,^34,35^ there are more than 3000 papers so far (June 2024) reporting the implementation of this reaction for mechanistic studies in plasmon chemistry.

Due to the long-term project of our group focused on mechanistic aspects of plasmon catalysis, we questioned the applicability of the azo coupling reaction of thiol-based SAMs as a model for mechanistic studies in the field of plasmon catalysis. The potential desorption of SAMs during plasmonic azo coupling has been hypothesized by Mahmoud^36^ but has not been confirmed experimentally. The potential unaccounted desorption of thiols during the plasmon excitation of NSs could lead to misinterpretation of the kinetic data and result in misleading conclusions for the field. To investigate this question, we chose typical spherical Au NPs assembled by PNTP deposited on a silicon substrate. We aimed at detailed tracking of the pathway of PNTP on the surface of Au NPs during plasmon excitation by X-ray photoelectron spectroscopy (XPS) to complement the Raman spectroscopy data. Further, we generated a kinetic model of plasmon-driven transformations of SAMs based on the experimental XPS data observations. The need for a plasmonic community in simple kinetic models for mechanistic studies inspired us to suggest an alternative reaction as a model, alkoxyamine homolysis, based on a simple evaluation of the wavelength and power of the irradiation effect using first-order kinetics.

## Results and Discussion

### Characterization of Au-NTP as a Model System

We commenced our investigation by preparing a commonly used substrate for the plasmon-induced reaction of azo coupling, as illustrated in Figure 1b. The substrate is composed of Au NPs with self-assembled PNTP layers (Au-NTP). Au NPs sized by 20 ± 2.8 nm (Figure S1) were prepared by the modified Turkevich method^37^ followed by functionalization by PNTP. The homogeneity of the Au NPs coating was confirmed by scanning electron microscopy (SEM) with energy-dispersive X-ray spectroscopy (EDX) mapping (Figure S2).

**Figure 1:**
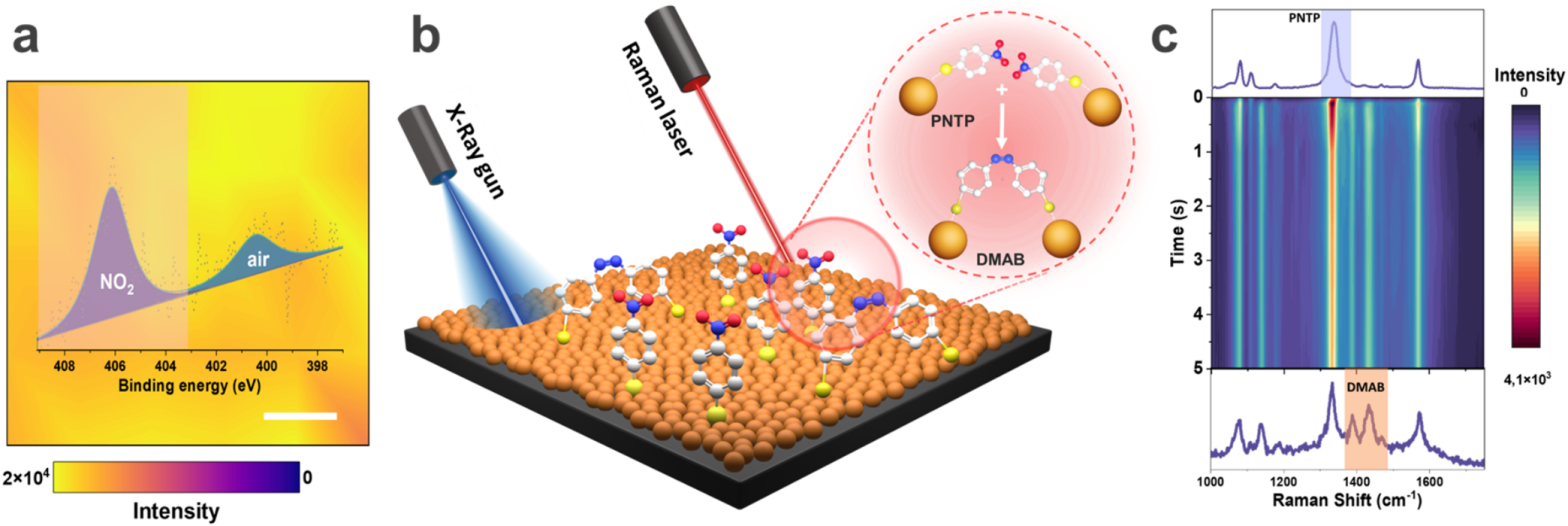
(a) XPS mapping image of the 406 eV peak intensity and the high-resolution spectrum of the N 1s region. Scale bar is 50 μm. (b) Schematic representation of the model substrate consisting of 20 nm Au NPs with PNTP deposited on silicon for the further study by Raman spectroscopy and XPS. (c) Time-resolved SERS mapping (0.1; 0.2; 0.5; 1; 2.5; 5 s step) and snapshots of SERS spectra (above and below) during plasmon-driven PNTP azo coupling (633 nm, 3 kW/cm^2^, 1s).

The formation of PNTP SAMs was confirmed by Raman spectroscopy and XPS. The Raman spectra presented in Figures 1c and S2 show characteristic peaks of Au-NTP, such as Au–S at 330 cm^–1^, C–S at 720 cm^–1^, C–N at 1074 cm^–1^, and –NO_2_ at 1330 cm^–1^, similar to previously published works^38,39^ (Table S1). The homogeneous distribution of PNTP was confirmed by the signal deviation of 14% for the intensity of the NO_2_ signal at 1330 cm^–1^ over a 250 × 250 μm^2^ area (Figure S2). Chemical states of sulfur and nitrogen on the surface were identified by XPS after preliminary optimization of measurement parameters (Figure S3, SI Note 1), where the high-resolution spectra of S 2p and N 1s regions correspond to the bond formation between thiol and gold (Au–S signal at 162 eV) and the presence of the nitro group from PNTP on the surface (NO_2_ signal at 406 eV) (Table S2, Figures 1a, S4 and S5, SI Note 2). The XPS study revealed the presence of the dinitrogen-related peak at 400.9 eV (Figure S6, Table S2 and SI Note 3) from air and transfer lines in the XPS setup, as reported elsewhere.^40,41^ Further experiments were carried out, considering the constant value of the background peak at 400.9 eV with a fixed area (SI Note 3). According to the attenuation of the Au 4f signal, the surface coverage was found to be 9.5 mol/nm^2^ (SI Note 4). XPS mapping for the NO_2_ signal (406 eV) over a 1000 × 1000 μm^2^ area shows the homogeneous distribution of PNTP across the surface with 8% deviation (Figure 1a).

### Raman and XPS Monitoring of Azo Coupling under Different Irradiation Powers

The prepared Au-NTP substrate was irradiated by a 633 nm laser installed in the Raman spectrometer with different power densities (10, 20, 40, 80 kW/cm^2^) for 5 s (Figure S7). The laser power range has been chosen according to the previous studies for the evaluation of the pH (64 kW/cm^2^),^15^ temperature (127 kW/cm^2^),^42^ reaction gas media (160 kW/cm^2^),^6^ and NSs of the plasmon-active substrates (0.8–17 kW/cm^2^).^21^ Based on the previous reports summarized in Table S3, we additionally test a longer reaction time of 60 s (Figure S8). The obtained Raman spectra clearly demonstrate the formation of DMAB by increasing the intensity of signals from ν­(C–N) and β­(CH) at 1135 cm^–1^ and ν­(N=N) at 1391 and 1430 cm^–1^ and decreasing the intensity of NO_2_ signals at 1075 and 1330 cm^–1^.^38,39,43^ The overall kinetic data (Figure S9, Table S4 and SI Note 5) confirm the successful azo coupling reaction, where the initial rates calculated from the linear range (without reaction order identification) are increasing with the laser power growth, similar to previously published trends.^7,34,44,45^ According to the analysis of these reports, the resulting kinetic dependencies were fitted using different equations, from pseudo-1st-order on spherical Au NPs^7^ to second-order on Au nanoflowers.^46^ The variety of kinetic data (according to many reports^7,44,47,48^ ) calls into question the applicability of the azo coupling reaction as the model one due to the difficulties in comparison between different substrates.

To shed light on the underlying process of PNTP coupling on plasmonic NPs during and after the reaction, the proper analytical tool must be chosen. Raman spectroscopy lacks the ability to differentiate similar chemical states. While transformation of NO_2_ to N=N is easy to monitor by surface-enhanced Raman spectroscopy (SERS) due to the high sensitivity, peaks observed for S–H, C–S, S–O, and Au–S bonds are low-intensive (Figure S10) and hard to detect due to the relatively weak polarizability of these bonds.^49,50^ Even so, the kinetic monitoring of Au–S (at 330 cm^–1^) and C–S (at 720 cm^–1^) Raman bands shows a decrease of intensity during azo coupling (Figure S11, SI Note 6). However, the low intensity of those signals makes it impossible to conclude about transformations of the thiol group (SI Note 6), including potential desorption processes. On the contrary, XPS surface sensitivity within a nanometer-scale depth aligns perfectly with the interfacial regions where the PNTP reduction occurs.^51,52^ This makes XPS an excellent tool for the evaluation of plasmon-driven catalytic reactions giving crucial information about the reaction kinetics and chemical stucture of the intermediates.

To study the surface processes during plasmon-induced dimerization of self-assembled PNTP by XPS, we had to consider a mismatch in the analytical techniques: a 200 × 100 μm^2^ spot size is required for XPS (Nexsa G2 Surface Analysis System) to get a reliable signal. Therefore, Au-NTP was exposed to Raman laser (10, 20, 40, 80 kW/cm^2^) in a mapping mode with a 250 × 250 μm^2^ area at 2500 points (≈2.67 μm laser spot) for 5 s each for further XPS analysis (Figures 2a–c and S12).

**Figure 2:**
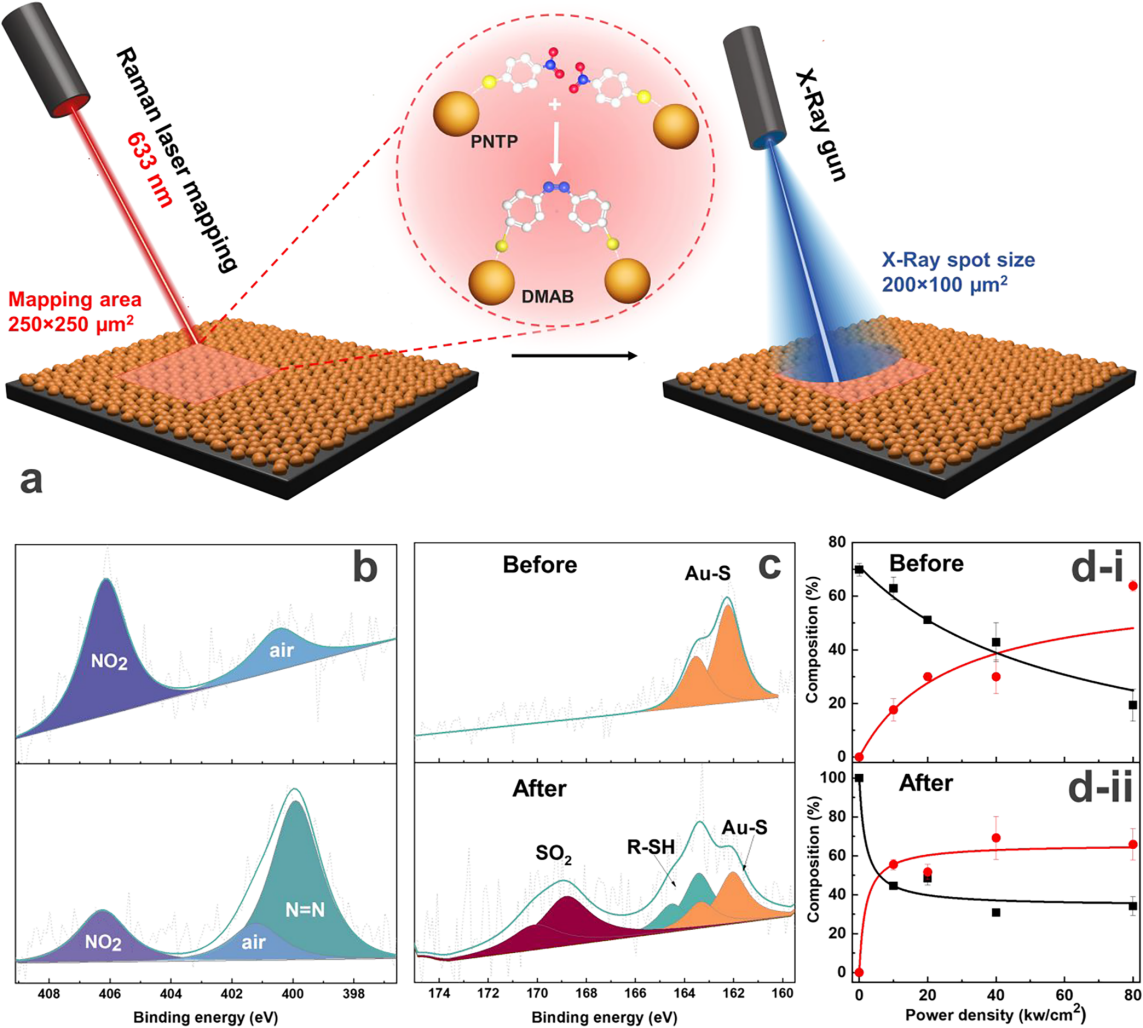
(a) Experimental strategy for detailed XPS study of the plasmon-induced azo coupling reaction, where Au-NTP deposited to the silicon substrate was treated by Raman laser (633 nm, 10–80 kW/cm^2^) in a mapping mode (250 × 250 μm^2^ or 50 × 50 points) for the creation of an area required for reliable XPS analysis with a 200 × 100 μm^2^ spot (the data represents the mean value with the standard deviation (SD) calculated from 3 measurements on 3 different spots (*N* = 3, *n* = 3)). (b) XPS spectra of the N 1s region obtained before and after the reaction. (c) XPS spectra of the S 2p region obtained before and after the reaction at 80 kW/cm^2^. (d) Kinetic curves of the azo coupling reaction tracked by changes of (i) the N 1s region, where red dots represent N=N at 400.2 eV and black squares represent NO_2_ at 406 eV and (ii) the S 2p region, where red dots represent SO_2_ at 168.7 eV and black squares represent Au–S at 162 eV.

For the reliable XPS analysis, we generated the data set of the reference XPS spectra of PNTP , 4-nitroaniline, and 4,4′-diaminoazobenzene powders, which highlights the major peaks of interest: NO_2_-related nitrogen, NH_2_, N=N, *N*_air_, and sulfur, related to various bond types (S–Au, S–H, *S*_ox_) (Table S2 and Figure S5). Initial Au-NTP shows 2 peaks in the N 1s region: the background at 400.9 eV and the NO_2_ signal at 406 eV (Figure 2b); the intensities of these peaks were used as references for further calculations (considering an 8% signal deviation shown in Figure 1a). After laser irradiation, the N 1s spectra show the formation of DMAB through an increase of the N=N signal at 400.2 eV, while the –NO_2_ (PNTP) signal at 406 eV decreased (Figure 2d–i). The changes in the N 1s region confirm the successful reaction with 6.5, 35.9, 68.2, and 76.6% conversion of NO_2_ for laser powers of 10, 20, 40, and 80 kW/cm^2^, respectively. Comparing the XPS-derived and Raman spectroscopy-obtained conversions of surface groups (4.4, 10.4, 24.5, 86.3%, respectively), some extent of discrepancy with the conservation of the overall trend is observed.

The high-resolution spectra of S 2p assisted in determining the state of sulfur before and after 5 s of laser irradiation (Figure 2c,d-ii), while Au-NTP shows a doublet at 162.2 and 163.5 eV referred to thiol Au–S bonds (Figure 2c). After laser irradiation, we observe a decrease in the Au–S-related peak and an appearance of two new doublets at 164.4, 165.5 eV and 168.7, 170.2 eV (Figure 2c). The first peak is assigned to S–H according to the measurement of the PNTP powder (Figure S5 and Table S2), while the second peak is assigned to oxidized thiol groups. The appearance of these peaks suggests that the side processes occurred simultaneously with the azo coupling reaction, such as thiol desorption and oxidation.

### XPS Monitoring of Reaction Kinetics to Propose Tentative Reaction Pathways and Kinetic Models

To monitor changes in the N 1s and S 2p regions in detail, we performed a kinetic investigation of dimerization at 80 kW/cm^2^ (Figures 3a,b and S13) and at 22 kW/cm^2^ (Figures 3c,d and S14) using the measurement intervals similar to Raman monitoring of the reaction (Figures S8 and S9). We plotted the ratio of the peak area of the azo product to pristine PNTP NO_2_/(NO_2_ + N=N) to confirm the successful reaction (Figure 3). According to Figure 3a,c, the peak at 406 eV gradually decreases with the growth of the peak at 400.2 eV. In turn, the ratio of the area under peaks of desorbed thiol to the pristine material, *S*_ox_/(SH + *S*_ox_ + Au–S), was applied to observe desorption and oxidation (Figure 3b,d), where the initial doublets at 162.2 and 163.5 eV related to Au–S decrease and the new doublets at 164.4, 165.5 eV (SH)^53^ and 168.7, 170.2 eV (*S*_ox_)^54^ appear and grow over the irradiation time almost simultaneously. Both NO_2_/(NO_2_ + N=N) and *S*_ox_/(SH + *S*_ox_ + Au–S) reach a plateau after a while, which can be explained by the limited ability to adopt favorable configurations for azo coupling-related products or to find a close enough neighboring and further transformation because of steric hindrance^55^ affording typically 10–70% NTP conversion.^56^ The observed spectral changes obtained with laser powers of 80 and 22 kW/cm^2^ are similar and do not depend on the applied power and reaction time.

**Figure 3:**
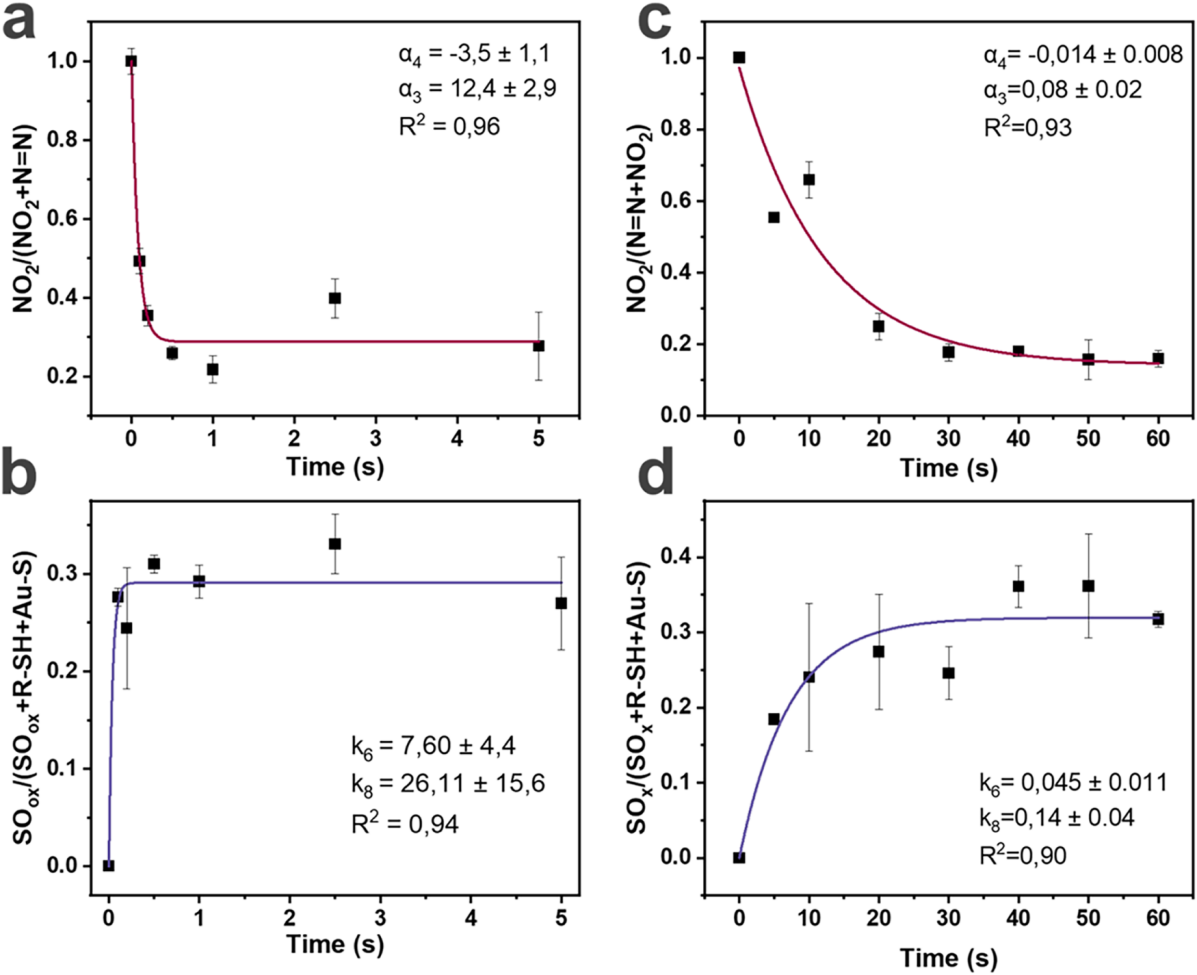
(a) Kinetic curve^[a]^ of NO_2_/(N=N + NO_2_) intensities vs time at 80 kW/cm^2^ and its fitting^[b]^. (b) Kinetic curve^[a]^ of *S*_ox_/(Au–S + SH + *S*_ox_) intensities vs time at 80 kW/cm^2^ and its fitting^[c]^. (c) Kinetic curve^[a]^ of NO_2_/(N=N + NO_2_) intensities vs time at 22 kW/cm^2^ and its fitting^[b]^. (d) Kinetic curve^[a]^ of *S*_ox_/(Au–S + SH + *S*_ox_) intensities vs time at 22 kW/cm^2^ and its fitting^[c]^; ^[a]^the data represents the mean value with the SD calculated from 3 measurements on 3 different spots (*N* = 3)^[b]^. Fitting was performed from the calculated eq 6 $\left[A\right]=\left({\left[A\right]}_{0}+\frac{\alpha_{4}}{\alpha_{3}}\right)e^{-\alpha_{3}t}-\frac{\alpha_{4}}{\alpha_{3}}$ according to Scheme 1 ^[c]^. Fitting was performed from the calculated eq 10 $\left[E\right]=\frac{k_{6}}{k_{8}}\left(1-e^{-k_{8}t}\right)$ according to Scheme 1.

The monitoring of reaction kinetics allowed us to suggest a plausible pathway, where initial PNTP (**A**) can generate target DMAB (**G**) with *k*_1_ (Scheme 1). Simultaneously, PNTP can be desorbed (**B**) from Au with *k*_2_ with a range of further transformations (*k*_3_) and be in the equilibrium with **A** (*k*_*‑2*_).^25^ Desorbed **B** in equilibrium with **A** species still can generate azo species **C** with *k*_4_, where one thiol is bonded to Au. The **C** species can again desorb forming **D** with *k*_5_. Alternatively, free-standing SH groups in **C** species can undergo oxidation, as displayed on Scheme 1, to **E** with *k*_6_. Again, bonded thiol in **E** can desorb affording **F** with *k*_8_. As thiols are in equilibrium with Au, the **C** species can be again generated from **G** with *k*_7_. Despite our hypothesis being quite complicated chemical pathways, there are a few alternative options, e.g., the oxidation of **D** species.

**Scheme 1:**
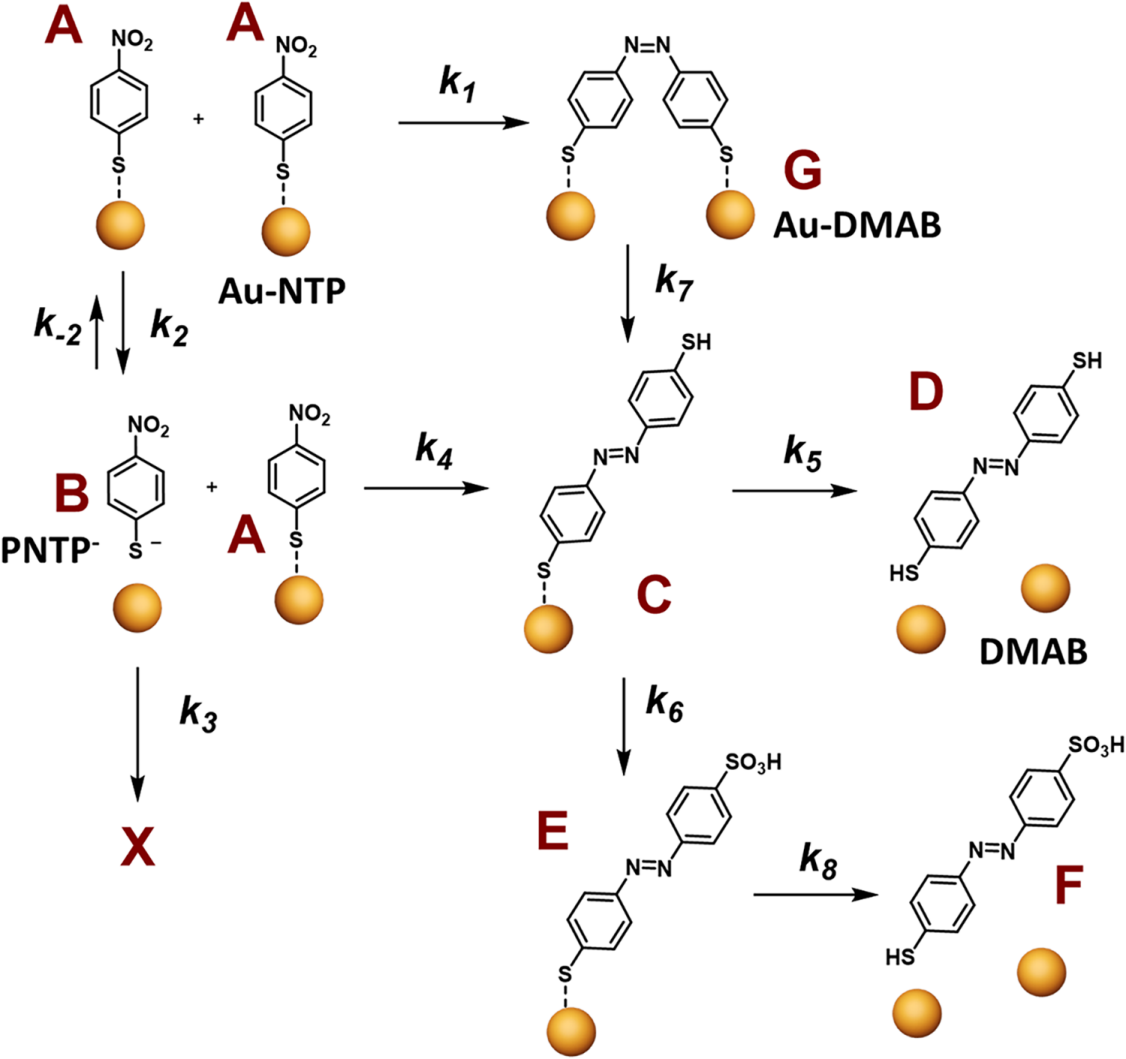
Tentative Chemical Pathway during Plasmon-Induced Azo Coupling Hypothesized Based on Obtained XPS Data

To fit the experimental data, we initially used standard approximation functions commonly applied for 0^th^, first/pseudo-1^st^, and second orders^4,7,45^ for the azo coupling reaction. However, these models resulted in poor fits, with *R* ^2^ < 0.2, indicating that they could not adequately describe the reaction. Then, we attempted the fractional lifetime method (comparing the times required to reach specific fractional conversions) to determine the reaction order for each process (Figure S15, SI Note 7). Unfortunately, this approach also failed due to the complexity of the reaction pathways. Therefore, our ultimate goal was to come closer to a more realistic and proper description of kinetic processes occurring during azo coupling. There are at least 8 possible reactions occuring (Scheme 1) with *k*_1–8_ and each reaction could have different kinetic orders giving more than thousand options of the overall kinetic model. Additional complexity is brought by the multistep character of **A** → **G**, **B** → **C**, and **C** → **E**.

We build our models considering the changes in NO_2_ (**A**), N=N compounds (**G, C, D, E**), Au–S bonds (**A, G, C, E**), and oxidized sulfur SO_*x*_ (**E**). Since XPS signals often represent multiple molecular species (e.g., the N=N signal corresponds to **C**, **G**, and **E**), the kinetic models will be designed to account for these overlapping contributions, grouping them under single XPS-detected signals. To reduce the number of possible kinetic models, we had to make a few assumptions, which are comprehensively described in SI Note 8. After implementing those assumptions, we had 8 reactions (**A** → **G**, **A** → **B**, **B** → **X**, **B** → **C**, **C** → **D**, **C** → **E**, **G** → **C**, **E** → **F)**, as shown in SI Note 9 and 10, where we varied the reaction order for 4 out of 8: **A** → **G** (assuming orders 0^th^, first, and second) and **B** → **A**, **B** → **C**, **C** → **E** (assuming orders 0^th^ and first), leading to kinetic schemes to be investigated and summarized in Table 1 (remaining reactions were assumed to be of first order).

**Table 1:**
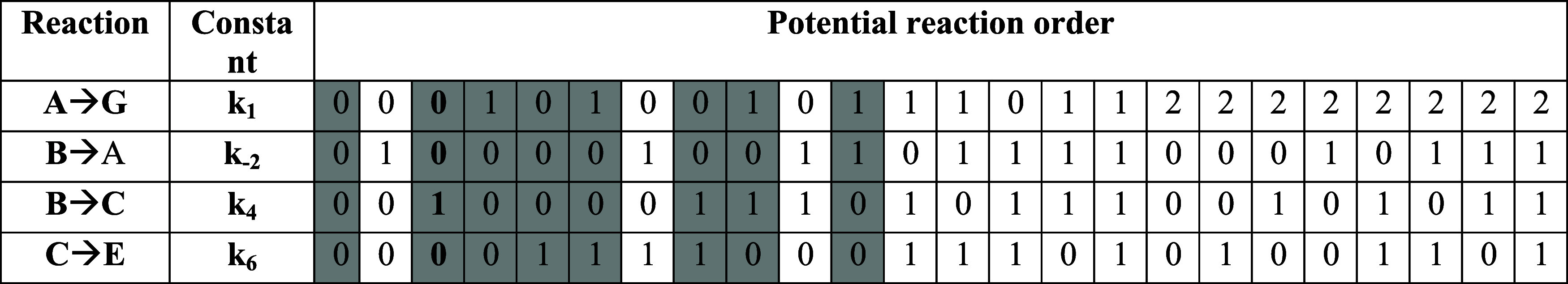
Overview for Kinetic Schemes for Plasmonic PNTP Azo Coupling Based on Chemical Pathways (Scheme 1) with the Most Important Reactions Assuming Different Orders^a^

a Columns colored in gray are the most promising combination.

To simplify the equations, we grouped related reaction rates *k_i_* into combined constants α_i_, allowing us to represent the reactions more generally (Table S5). This approach helped us narrow the possible kinetic pathways to a manageable number while still capturing the key trends in the experimental data. For observed species, several general equations are available as displayed for the model assuming first order for the fragmentation:

1 $$\alpha_{i}t+\sum \exp\left(\mathrm{for}\left[\mathrm{Au}\text{−}S\right],\;\mathrm{and}\;\left[N=N\right]\right)$$

2 $$\left({\left[A\right]}_{0}+\alpha_{i}/\alpha_{j}\right)e^{-\alpha j\cdot t}+\alpha_{i}/\alpha_{j}$$

where α_*i* or *j*_ is the combination of rate constants; exp is the exponential function; *t* is the time; and [*A*]_0_ is the initial concentration of the appropriate compound.

From Table 1, it can be observed that only the models highlighted in gray were consistent with Scheme 1. Alternative scenarios are shown in SI Note 9 and Figure S16. Next, kinetic equations are developed for the model pointed out as bold symbols listed in Table 1, taking into account the corresponding assumptions (SI Note 8) (reaction­(order)): *k*_1_(0^th^), *k*_2_(1st), *k*_–2_(0^th^), *k*_3_(1st), *k*_4_(1st), *k*_5_(1st), *k*_6_(0^th^), *k*_7_(1st), and *k*_8_(1st). The solutions are described in Table 2.

**Table 2:** Differential and Integrated Forms of Equations Describing a Kinetic Model of Plasmon-Induced Azo Coupling

differential form	integrated form
3 $\frac{d\left[B\right]}{dt}=0=k_{2}\left[A\right]-k_{-2}-k_{3}\left[B\right]-k_{4}\left[B\right]$	4 $\left[B\right]=\frac{k_{2}}{k_{3}+k_{4}}\left[A\right]-\frac{k_{-2}}{k_{3}+k_{4}}=\alpha_{1}\left[A\right]-\alpha_{2}$
5 $-\frac{d\left[A\right]}{dt}=k_{2}\left[A\right]-k_{-2}+k_{4}\left[B\right]+k_{1}$	6 $\left[A\right]=\left({\left[A\right]}_{0}+\frac{\alpha_{4}}{\alpha_{3}}\right)e^{-\alpha_{3}t}-\frac{\alpha_{4}}{\alpha_{3}}$ ^a^
7 $-\frac{d\left[G\right]}{dt}=k_{7}\left[G\right]-k_{1}$	8 $\left[G\right]=\frac{k_{1}}{k_{7}}\left(1-e^{-k_{7}t}\right)=\alpha_{5}\left(1-e^{-k_{7}t}\right)$
9 $\frac{d\left[E\right]}{dt}=k_{6}-k_{8}\left[E\right]$	10 $\left[E\right]=\frac{k_{6}}{k_{8}}\left(1-e^{-k_{8}t}\right)=\alpha_{6}\left(1-e^{-k_{8}t}\right)$
11 $\frac{d\left[C\right]}{dt}=k_{4}\left[B\right]+k_{7}\left[G\right]-k_{5}\left[C\right]-k_{6}$	
Integrated form of eq 11:	
12 $\left[C\right]=\alpha_{9}\left(e^{-\alpha_{3}t}-e^{-k_{5}t}\right)-\alpha_{10}\left(e^{-k_{7}t}-e^{-k_{5}t}\right)+\alpha_{11}\left(1-e^{-k_{5}t}\right)=\alpha_{12}e^{-k_{5}t}+\alpha_{9}e^{-\alpha_{3}t}-\alpha_{10}e^{-k_{7}t}+\alpha_{11}$ ^b^,^c^	

a Where $\alpha_{3}=k_{2}+k_{4}\alpha_{1}=k_{2}+\frac{k_{4}k_{2}}{k_{3}+k_{4}}>0$ (eq 6a); $\alpha_{4}=k_{1}-k_{-2}-k_{4}\alpha_{2}=k_{1}-k_{-2}-\frac{{k_{4}k}_{-2}}{k_{3}+k_{4}}<0$ (eq 6b); and $k_{1}<k_{-2}\left(\frac{{{2k}_{4}+k}_{3}}{k_{3}+k_{4}}\right)$ (eq 6c).

b Where $\alpha_{3}=\frac{{k_{2}k_{3}+2k}_{4}k_{2}}{k_{3}+k_{4}}$ and $\alpha_{4}=\frac{k_{1}\left(k_{3}+k_{4}\right)-k_{-2}k_{3}-2{k_{4}k}_{-2}}{k_{3}+k_{4}}$ , then $\frac{\alpha_{4}}{\alpha_{3}}=\frac{k_{1}\left(k_{3}+k_{4}\right)-k_{-2}\left(k_{3}+2k_{4}\right)}{k_{2}\left(k_{3}+2k_{4}\right)}$ and $\frac{{\alpha_{1}\alpha }_{4}}{\alpha_{3}}=\frac{k_{2}}{k_{3}+k_{4}}\left(\frac{k_{1}\left(k_{3}+k_{4}\right)-k_{-2}\left(k_{3}+2k_{4}\right)}{k_{2}\left(k_{3}+2k_{4}\right)}\right)$.

c From (eq 12), *t* → ∞, [*C*]_∞_ = α_11_ ≥ 0, it involves $k_{6}\leq \frac{k_{1}\left(k_{3}+k_{4}\right)}{k_{3}+2k_{4}}$ (eq 13) and implementing (eq 6b) in (eq 13), it gives *k*_6_ ≤ *k*_–2_.

As all *k_i_* values are positive, we required −α_4_/α_3_ > 0. Since α_1_ and α_2_ are always positive (SI Note 10), this condition was satisfied by setting α_3_ > 0 and α_4_ < 0. Based on this, we developed the kinetics for other species and identified valid models highlighted in gray, as listed in Table 1, corresponding to Scheme 1. The selected model describes species **C**, **G**, and **E** with reaction orders *k*_6_ (0^th^ order), *k*_1_ (0^th^ order), *k*_4_ (1st order), and *k*_–2_ (0^th^ order), as detailed in eqs 12, 8, and 9 (Tables 1 and 2).

As a straightforward solution, we focused on one model, verified on species **C**, and tested it by calculating α*_i_* values at *t*_0_ or *t*_∞_. This yielded meaningful results for **N=N** and **SH** species (eqs 12, 13, 14):

14 $$\begin{array}{l} \begin{array}{lll} \left[{\mathrm{NO}}_{2}\right]=\left[A\right] & \left[{\mathrm{SO}}_{2}\right]=\left[E\right] & \left[\mathrm{SH}\right]=\left[C\right] \end{array}\\ \left[N=N\right]=\left[G\right]+\left[C\right]+\left[E\right] \end{array}$$

For [N=N], when *t* → ∞, [N=N]_∞_ = α_14_ > 0 (eq 15) is fulfilled when (eq 16) hold.

15 $${\left[N=N\right]=\alpha }_{5}-\alpha_{5}e^{-k_{7}t}+\alpha_{12}e^{-k_{5}t}+\alpha_{9}e^{-\alpha_{3}t}-\alpha_{10}e^{-k_{7}t}+\alpha_{11}+\alpha_{6}-\alpha_{6}e^{-k_{8}t}$$

16 $${\left[N=N\right]=\alpha }_{12}e^{-k_{5}t}+\alpha_{9}e^{-\alpha_{3}t}-\alpha_{13}e^{-k_{7}t}-\alpha_{6}e^{-k_{8}t}+\alpha_{14}$$

17 $$k_{1}>\frac{k_{6}k_{7}\left(k_{3}+{2k}_{4}\right)\left(k_{8}-k_{5}\right)}{k_{8}\left(k_{5}\left(k_{3}+2k_{4}\right)+k_{7}\left(k_{3}+k_{4}\right)\right)}$$

18 [Au−S]=[A]+[G]+[C]+[E]

19 $$\left[\mathrm{Au}\text{−}S\right]=\alpha_{15}t+\alpha_{6}e^{-k_{8}t}+\alpha_{14}e^{-k_{7}t}-\alpha_{18}e^{-\alpha_{3}t}-\alpha_{12}e^{-k_{5}t}+\alpha_{16}$$

The model equations listed in Table 2 accurately captured the experimental data (Figure 3) for both kinetic study cases: 80 kW/cm^2^, 5 s: *R* ^2^ > 0.94 and 22 kW/cm^2^, 60 s: *R* ^2^ > 0.9 (Figure 3b). The high convergence of experimental data with the model presented in Table 2 confirms the kinetic validity of the model. Importantly, the reaction **B** → **A** was determined to be zeroth order for successful model solutions; therefore, other models were excluded.

To sum up, the values of the fitting parameters are not compared to the data provided in the literature as they are combinations of *k_i_*, which do not always describe the events reported in the literature. The analysis revealed that the kinetics of plasmon-induced PNTP azo coupling cannot be explained by simple orders (zeroth, first, second, or third)^4,44,57^ as were previously reported. Instead, a combination of exponential equations listed in Table 2 is required to accurately capture the reaction’s complexity. This divergence highlights the limitations of simplified models and their potential to lead to unreliable conclusions in fundamental studies.

### Plausible Mechanism of Thiol Desorption

Experimental evidence obtained from the XPS study prompted us to question the reason for thiol desorption during azo coupling. There are three commonly accepted mechanistic pathways in plasmon catalysis, namely, direct transfer of hot carriers (electrons or holes), intermolecular excitation, and plasmonic heating.^8,58^ There are debates focused on the elucidation of the mechanism for plasmonic azo coupling of PNTP, where most studies discuss hot electron transfer^45,59^ and plasmonic heating.^46,60,61^ Analysis of the local density of states (LDOS) of adsorbed PNTP molecules by density functional theory (DFT) calculations indicated that an energy of 1.95 eV (633 nm) is sufficient to excite absorbed PNTP (SI Note 11, Figure S17). This agrees with the generally considered thiolate–Au bond energy of 200 kJ/mol or 2 eV^62^ (SI Note 11).

Therefore, we do not exclude charge transfer excitation or chemical interface damping but also consider the possibility of plasmonic heating. Taking into account the long established SAM dissociation under the elevated temperatures,^27–29^ the plasmonic heating could also be responsible for desorption. Previous investigations of the plasmonic reaction in the solid phase by Sivan et al.^63^ and Linic et al.^64^ showed that the local temperature rise could reach over 100 °C. To evaluate the possibility of this scenario, we experimentally determined the temperature required to desorb PNTP in the air using thermogravimetric analysis (TGA). TGA results (Figures S18, S19, SI Note 12) showed that in the case of local heating over 223 °C, thiol desorption may occur. To evaluate the plasmonic heating, we estimated the local temperature (*T*_heat_) rise by numerical calculations (SI Note 13, Figure S20) and compared the reaction progress at 25 and 100 °C (Figures 4, S21, SI Note 14).

**Figure 4:**
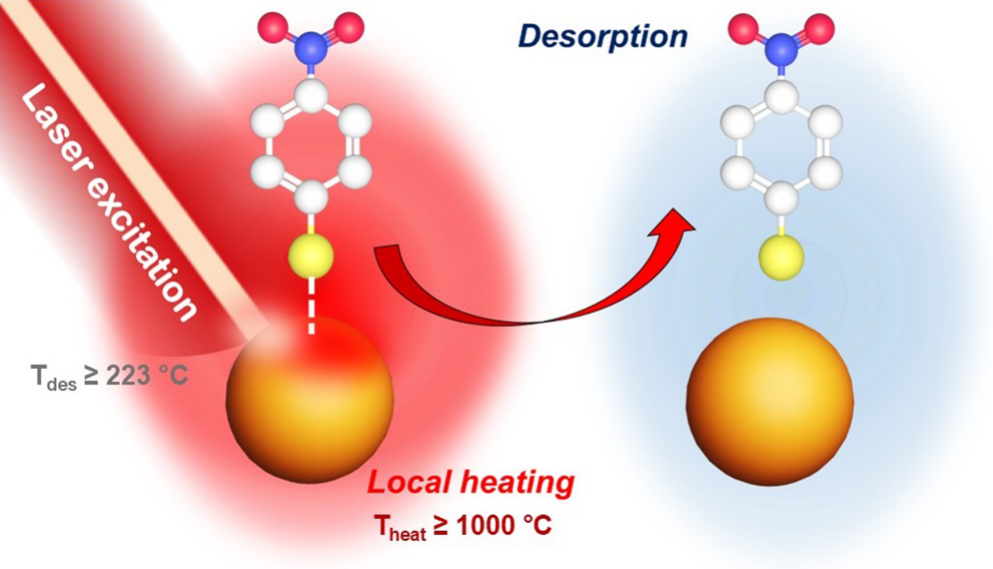
Thiol desorption through local heating.

The numerical simulations according to Sivan et al.^63^ assisted in the evaluation of *T*_heat_ at different laser powers. The laser power-dependent calculations show that *T*_heat_ is higher than *T*_des_ for the laser power >20 kW/cm^2^ and can reach over 1000 °C (Table S6). As a result, the *T*_heat_ was found to be over *T*_des_ = 223 °C, hypothesizing that plasmonic heating is responsible for thiol desorption.

According to previous reports, if plasmonic heating occurs, the reaction is intensified at higher temperatures.^42,61,65^ Additionally, we compared the azo coupling reaction (14 kW/cm^2^, 60 s irradiation) at 25 and 100 °C (below *T*_des_ = 223 °C). The analysis recorded changes of N=N, NO_2_, Au–S, and C–S Raman peaks (SI Note 14), which showed an increase in N=N and a decrease in other peaks at 100 °C compared to 25 °C. The intensities of S-related bonds also showed a decrease, suggesting partial desorption. This trend is consistent with previous studies,^42,61,65^ which demonstrated that localized plasmonic heating can enhance the azo coupling efficiency. The results, as presented in Figure S21, confirm the effect of local heating on plasmon-initiated azo coupling, which may also contribute to thiol desorption.

The crucial contribution of plasmonic heating was previously reported by the numerical finite element solver,^60^ Raman thermometry,^42,58^ and power-dependent experiments,^66^ comparing plasmonic and external heating.^46,61^ Therefore, previous reports and our numerical calculations, complemented by temperature-dependent Raman experiments, show that the *T*_heat_ is higher than the *T*_des_, concluding that the plasmonic heating is the main driving force of thiol desorption during azo coupling of PNTP on Au NPs.

### Alternative Model Reactions beyond NTP Azo Coupling

Considering the paradigm applicability of azo coupling as a model reaction in plasmonic chemistry, the question of the ideal model reaction arises. The ideal model reaction should be a simple transformation with known products and predictable and with well-studied reactivity.^67^ Moreover, the progress of the reaction and kinetics should be monitored by simple and precise detection/analysis methods. Azo coupling is monitored by Raman spectroscopy, which is a convenient method; however, it fits only for surface reactions, where the exact structure of products is challenging to establish.^68^ Our integrative study by XPS showed that the reactivity of PNTP on Au NPs is affected by thiol desorption; therefore, the full scope of products cannot be determined with high accuracy by Raman spectroscopy. The suggested kinetic model in eqs 6 and 10 based on >8 reactions, as shown in Scheme 1, indicates that the reaction order cannot be clearly fitted with 0^th^, first, or second orders. Therefore, the comparison of reaction rates during the investigation of the mechanism, effect of environment, irradiation mode, etc. cannot be precise.

Recently, we showed plasmon-induced homolysis of alkoxyamines (AAs)^8,9,69^ via intramolecular excitation as a model reaction to investigate plasmonic heating and the effect of the chemical structure on catalysis efficacy. This reaction proceeds with the generation of stable nitroxides (Figure 5a) easily monitored by electron paramagnetic resonance (EPR) spectroscopy. Homolysis processes represent first-order and one-stage transformations without additional reagents being an appealing option for the model reaction in plasmon catalysis.^8,9,69^ AAs could be homolyzed via plasmon excitation, generating reactive carbon-centered radicals and stable nitroxides; the concentration of the last could be easily and precisely defined with EPR spectroscopy (Figure 5a).^8,9,70^ The homolysis of AAs could proceed in bulk suspension mode^8^ and on-surface via preliminary modification via diazonium chemistry.^9^ Here, as an example, we show that AAs could be easily used as probe molecules to conduct fundamental studies on plasmonic nanostructures due to commercial availability.^71–73^ As an example of **SG1-St** used as a probe in a suspension of Au NPs, we show that the effect of the (i) excitation wavelength and (ii) power to the plasmonic performance of Au NPs could be easily studied.

**Figure 5:**
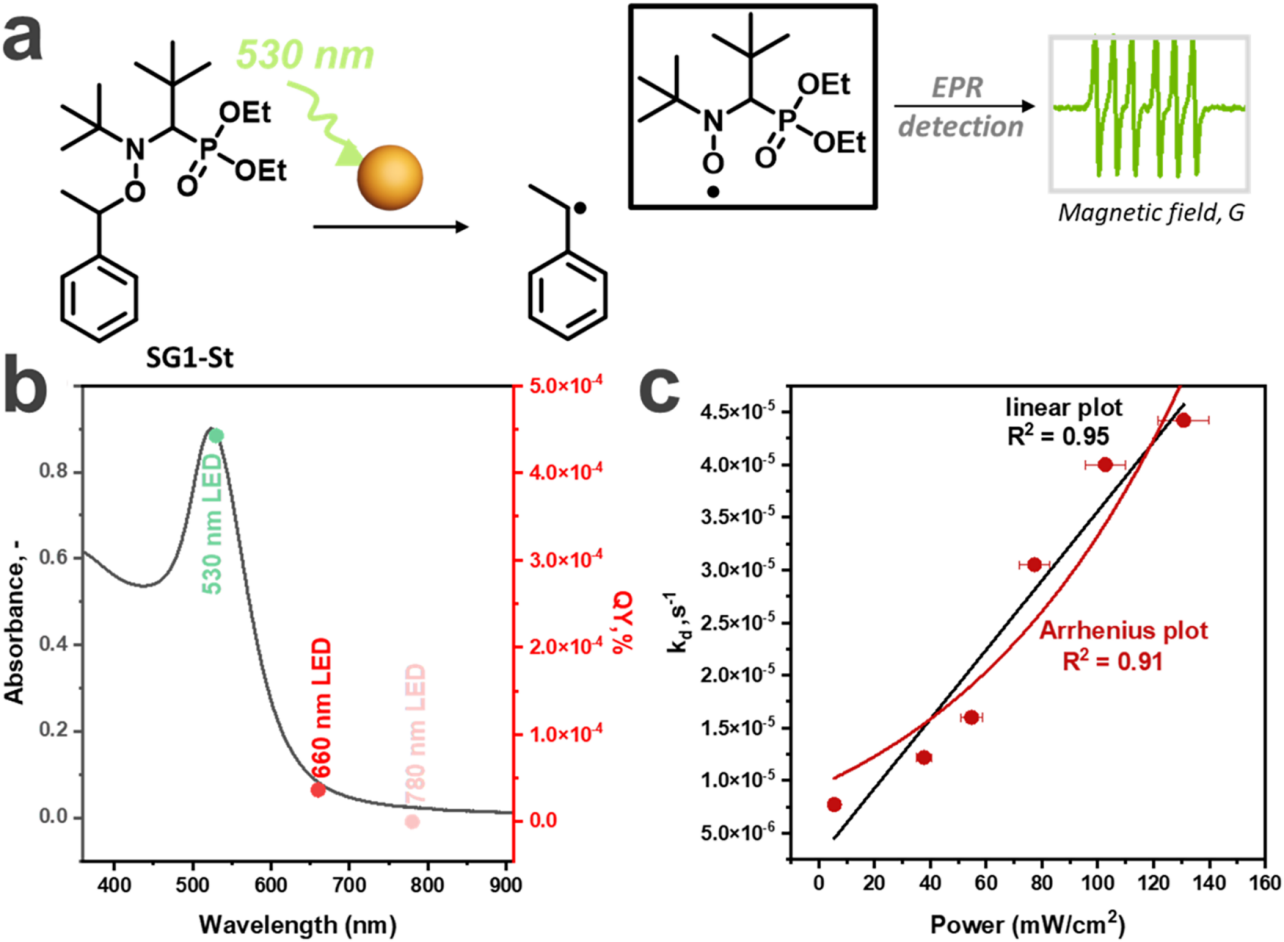
(a) Plasmon-driven C–ON bond homolysis of alkoxyamine **SG1-St** in solution. (b) UV–vis spectrum of Au NPs and the QY depicted as colored dots for plasmon-induced homolysis with 530 (green), 660 (red), and 780 (pink) nm light-emitting diodes (LEDs). (c) Correlation between *k*_d_ (s^–1^) of plasmon-induced homolysis and power of LED irradiation fitted with linear and Arrhenius functions (where A was fixed to be 2.4 × 10^14^ s^–1^).

We studied the effect of the irradiation wavelength on 13 nm Au NPs with a maximum of the plasmon resonance localized at 520 nm. Thus, we have examined 530, 660, and 780 nm LED irradiation to (i) confirm the plasmonic activity similar to previous reports^74–76^ and (ii) possibly distinguish the photochemical and photothermal nature by varying the power.^77^ The homolysis of **SG1-St** led to nitroxide SG1 concentration vs time dependence, which allowed us to plot kinetic curves, fitted with an exponential function, and calculate rate constants *k*_*d*_ (SI Note 15, Figures 5b, S22). Due to the difference in power of each light source, the quantum yields (QYs) were evaluated to unify the photon energy (Figure 5c, Table S7, SI Note 16). First, the larger QY was provided by a 530 nm LED that associated with the plasmon resonance maximum. Further increasing the wavelength led to a decrease in QYs due to the lack of overlapping between the emission of LEDs and plasmon resonance, which is in good agreement with previously reported data.^18^

Second, alkoxyamine homolysis may also be a probe to investigate the power dependency of the plasmon-initiated reaction to distinguish chemical and thermal effects. According to Baffou et al. and others,^77,78^ fitting of the power dependence of *k*_*d*_ to a linear or exponential function reveals the nature of the chemical process. So, we performed the C–ON bond cleavage of **SG1-St** in the presence of the same Au NPs at 530 nm at different powers and plotted kinetic curves (Figure S23, SI Note 17). Fitting with an exponential function that corresponds to the first-order reaction provided rate constants of the reaction at different powers. Fitting with an Arrhenius function could not be obvious because there are a few parameters (such as activation energy *E*_a_, frequency factor *A*, specifically for constant *C*), which could be fixed or nonfixed. We developed an online tool^79^ for fitting our data, where for the Arrhenius function, we fixed the A factor to be 2.4 × 10^14^ s^–1^ reflecting a homolysis type of reaction.^80,81^ Experimental data have to be fitted with the Arrhenius function to have a physical meaning for a specific system. The increasing power of irradiation augmented the efficacy of homolysis (Table S8), and the correlation between *k*_*d*_ and power had a linear shape (*R*_lin_^2^ = 0.95) and *R*_exp_^2^ = 0.91 for Arrhenius plot fitting. The linear fit correlation slightly dominates over Arrhenius; however, the inconsiderable difference suggests that some photothermal effects in addition to photoeffects could play a role in homolysis. AA homolysis could be further applied to verify and to investigate plasmon catalytic activity for newly synthesized substrates or compare plasmonic activity between different substrates using *k*_d_ or QY.

## Conclusions

Although azo coupling of PNTP was previously used for plasmonic studies with a hypothesized simple chemical pathway,^7,28,36,42,46,49,60,61,63,66,82^ here, we challenge the status quo of the model reaction. Our detailed XPS study revealed the partial desorption and oxidation of PNTP and related products during the reaction. N 1s and S 2p regions were collected on Au-NTP before and after azo coupling, including kinetic measurements. The XPS studies showed that (i) thiol desorption occurs in the laser power range of 10–80 kW/cm^2^ being common for previous reports^6,15,21,42^ and (ii) both azo coupling and desorption occur simultaneously and immediately after irradiation independent of the laser power. The kinetic XPS study hypothesizes tentative reaction pathways including more than 8 reactions (Figure 3c), where fitting of these reactions with zeroth, first, and second orders did not show high convergence in contrast to eqs 6, 9, and 10, showing the complexity of the real model. The low convergence with simple reaction orders used in the previous studies^7,34,45,46,82^ expresses doubt about the reliable evaluation of azo coupling kinetics. We developed a complex kinetic model consisting of a set of exponential equations, as listed in Table 2, with *R* ^2^ > 0.9. Moreover, Allesandri recently showed that azo coupling can proceed on TiO_2_, ZnO, and SiO_2_ without excitation of plasmons.^83^ Therefore, the origin of the reaction is debated not only between hot carrier transfer^45,59^ and plasmonic heating^46,60,61^ but also between laser-induced activation of singlet oxygen species^83^ without plasmons.

The azo coupling reaction does not fit within the requirements of the model one (Figure 6a), which should be simple and proceed via well-established mechanisms.^67^ For the model reaction, we should be able to exclude side reactions, and reaction rates are easily and quantitatively assessed for known products. However, the removal of DMAB or related products from the surface for quantification/product analysis is an elusive task.^68^ According to Camargo,^68^ typical indicators of the catalyst, such as the QY and/or turnover number/frequency cannot be calculated for azo coupling because reactant and product molecules are functionalized at the surface. Therefore, alternative reactions, such as oxidation,^64,84–86^ hydrogenation/water splitting,^87–89^ or homolysis^8,9^ in a bulk mode should be considered by the plasmonic chemistry community as model ones for the evaluation of fundamental parameters. Here, we showed that AA exemplified with **SG1-St** might be used as probe molecules in plasmon-driven reactions to evaluate the effect of different parameters (e.g., wavelength, power) for mechanistic study because of the simplicity of the reaction with known products and kinetic models (Figure 6b). These findings aim to draw the plasmonic community’s attention to model reactions for mechanistic studies, which is crucial for the further development of plasmonic chemistry.

**Figure 6:**
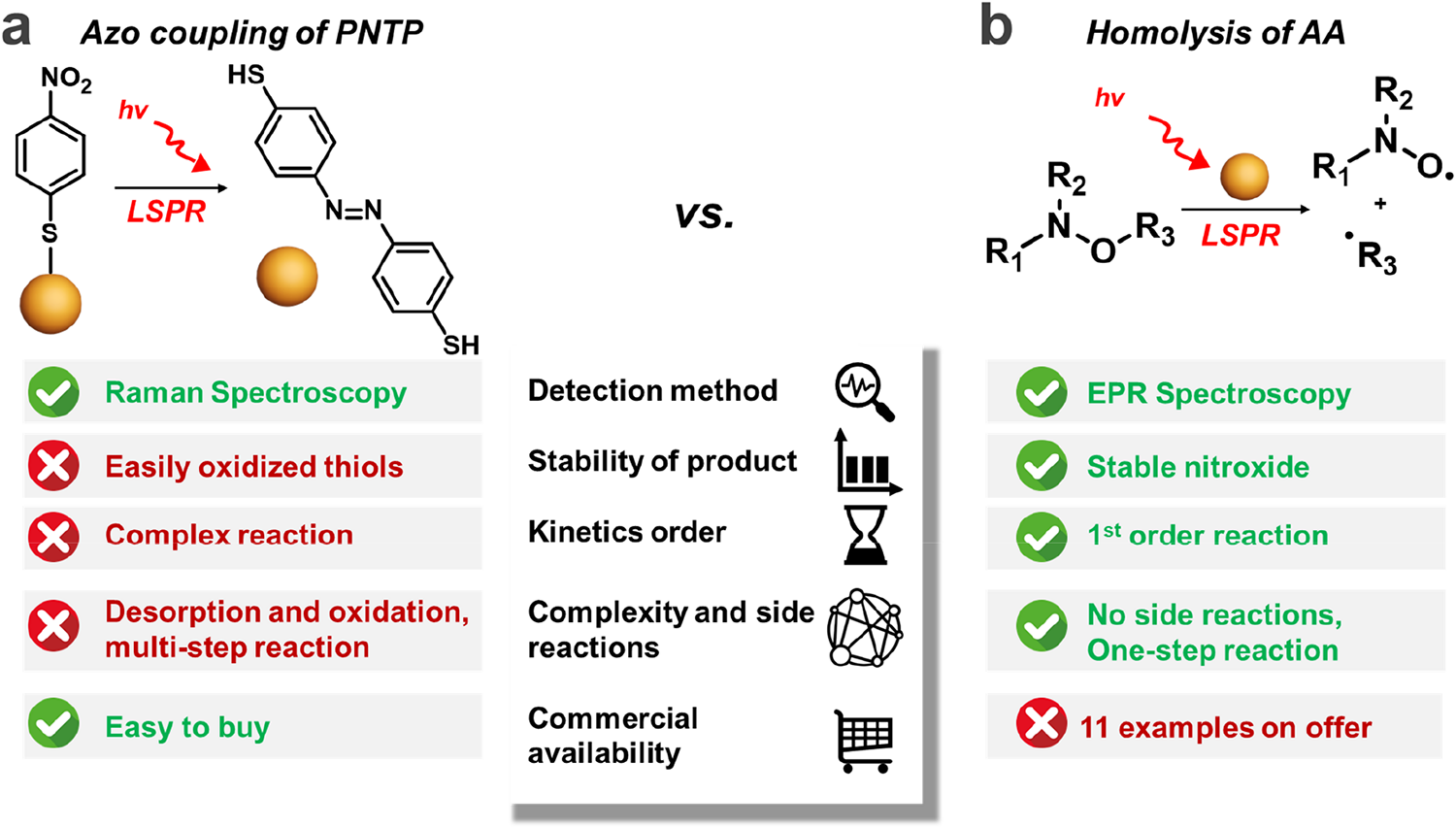
Comparison of (a) PNTP azo coupling and (b) AA homolysis as model reactions for plasmon catalysis fundamental studies.

## Materials and Methods

### Materials

All chemicals used were of analytical grade or of the highest purity available to us. HAuCl_4_ (99.995%), trisodium citrate dihydrate (Na_3_CA, >98%), ethanol, methanol (reagent grade, ≥99%), *p*-nitrothiophenol (technical grade, 80%), *p*-nitroaniline (≥99%), p-aminoazobenzene, and all other reagents for **SG1-St** preparation were purchased from Sigma-Aldrich and Alfa Aesar and used without further purification. All experiments were performed in deionized water. All glassware was thoroughly cleaned with freshly prepared 3:1 HCl/HNO_3_ (aqua regia) and rinsed thoroughly with water prior to use.

### Preparation of Au NPs

Gold nanoparticles (Au NPs) were prepared according to the modified Turkevich method.^1^ One mL portion of 0.025 M HAuCl_4_ solution and 2.5 mL of 0.038 M Na_3_CA solution in water were added to 100 mL of refluxing water under continuous stirring. The solution was heated for 10 min until the color changed to red. The resulted solution was cooled to room temperature and used as prepared.

### Modification of Au NPs by PNTP

Freshly prepared Au NP solution (40 mL) was mixed with 15 mM solution of PNTP in ethanol (5 mL) and stirred for 3 h. After modification, Au NPs were purified by centrifugation and washing with water/ethanol (1/3, 30 mL/20 mL, 7500 rpm, 30–60 min). After each centrifugation cycle, Au NPs were sonicated for 1–2 min. The resulting precipitate of Au NPs was resuspended in 50 μL of MeOH. 50 μL of the resultant Au-NTP solution was deposited on a silica slide (0.5 × 0.5 cm^2^) with a template by drop-casting for further XPS and Raman study.

### Azo Coupling Reaction Using Raman Laser

Time-resolved SERS spectra were collected by using a Renishaw inVia Raman microscope under a 633 nm continuous-wave (CW) laser in the confocal mode (focal area of 2.7 μm diameter). A 20× objective (NA = 0.5, WD = 10.6 mm, and an Olympus LMPLFLN-BD) was used for both plasmon-induced coupling and Raman signal collection. The plasmon-induced reaction was performed using the power density (10, 20, 22, 40, 80 kW/cm^2^) for 0.1, 0.5, 1, 2.5, 5, 10, 20, 30, 50, and 60 s in a mapping mode within the surface area of 250 × 250 μm^2^ providing 50 × 50 points. After irradiation, Raman signal collection was performed with an acquisition time of 1 s and a 3 kW/cm^2^ power. Raman spectra were recorded five times to estimate the standard deviation according to the following equation: $\mathrm{SD}=\sqrt{\sum \left|\frac{x-\mathrm{x̅}}{N}\right|}$ , where *x* is the value of the Raman signal intensity, *x̅* is the mean of the Raman signal intensity, and *N* = 5.

### Azo Coupling Reaction Using Raman Laser at 100 °C

The effect of heating on azo coupling was monitored by Raman spectroscopy using additional heating under 100 °C. The reaction was performed using LabRAM ARAMIS (Horiba, Japan) with red (633 nm) laser at a power density of 14 kW/cm^2^ for 60 s. Raman spectra were acquired with the same spectrometer with a power density of 3 kW/cm^2^ (without an influence on DMAB formation). The laser beam was focused on the sample using a 50× magnification objective (number of scans: 1, exposure time: 1 s).

### Preparation of Alkoxyamine **SG1-St**

Alkoxyamine **SG1-St** was synthesized according to the previously reported procedure via the atom transfer radical addition reaction.^2^

### Plasmon-Driven C–ON Bond Homolysis of **SG1-St** at LED Irradiation of Different Wavelengths

To 1.2 mL of the Au NP as-prepared suspension, a solution of alkoxyamine **SG1-St** in methanol (0.1 mM, 0.3 mL) was added. Finally, Milli-Q water was added to maintain the volume equal to 3 mL. The reaction mixture was stirred at room temperature and irradiated with an LED of appropriate wavelength located directly above the reaction vessel (2 cm).

### Plasmon-Driven C–ON Bond Homolysis of Alkoxyamine at LED Irradiation of Different Powers

To 1.2 mL of the Au NP as-prepared suspension, a solution of alkoxyamine **SG1-St** in methanol (0.1 mM, 0.3 mL) was added. Finally, Milli-Q water was added to maintain the volume equal to 3 mL. The reaction mixture was stirred at room temperature and irradiated with a 530 nm LED of appropriate power located directly above the reaction vessel (2 cm).

## Characterization Methods

### Scanning Electron Microscopy with Energy-Dispersive X-ray (SEM-EDX) Analysis

The images were taken on a Tescan MIRA 3 LMU instrument in reflected electron diffraction mode. The instrument was equipped with an Oxford Instrument Ultim Max 40 energy-dispersive X-ray spectroscope. SEM energy-dispersive X-ray spectroscopy (EDX) scanning was performed at an accelerating voltage (HV) of 20 kV.

### Transmission Electron Microscopy (TEM)

TEM observations were performed on a Philips CM 12 microscope.

### X-ray Photoelectron Spectroscopy (XPS)

The XPS spectra were recorded on a Thermo Fisher Scientific XPS NEXSA spectrometer equipped with an Al K α X-ray monochromatic emitter with an energy of 1486.6 eV. Survey spectra were recorded using a pass energy of 200 eV with an energy resolution of 1 eV. The spectra were calibrated against the C 1s peak set at 284.8 eV. Conditions for XPS measurements were chosen according to a preliminary test of X-ray irradiation damage on the Au–S stability. High-resolution spectra in the S 2p region of initial Au-NTP were recorded under different values of the pass energy: 50, 60, 70, 80, and 100 eV, with a resolution of 0.1 eV for each. For the high-resolution analysis of the area after Raman laser treatment in a mapping mode (250 × 250 μm^2^), the treated region was analyzed using a 100 × 200 μm^2^ scanned area with a flood gun for the charge compensation. High-resolution spectra (C 1s, N 1s, O 1s, S 2p, Au 4f) were collected using a pass energy of 50 eV and a resolution of 0.1 eV. The concentrations of elements were calculated in atom % using the sensitivity factors provided by the manufacturer. For the SD calculations of XPS, high-resolution spectra have been measured three times, and they were used with the SD calculated from N measurements according to the relation $\mathrm{SD}=\sqrt{\sum \left|\frac{x-\mathrm{x̅}}{N}\right|}$ , where x is the measured value, *x̅* is the mean of the measured values, and *N* = 3.

### Ultraviolet–Visible (UV–Vis) Spectroscopy

Adsorption spectra of the Au NP suspension were recorded using an Analytik Jena SPECORD250+ spectrometer in the 350–1100 nm range with a 200 nm/min scanning speed and a 1 nm resolution. Deionized water was used as a blank.

### Raman Spectroscopy

For the analysis of the chemical composition of the samples, Raman spectra were acquired using a Renishaw inVia Raman microscope with red (633 nm) laser sources with a power density of 3 kW/cm^2^ (without an influence on DMAB formation). The laser beam was focused on the sample using a 20× objective (number of scans: 1, exposure time: 1 s).

### Thermogravimetric Analysis (TGA)

TG analysis was carried out with a Q600 Simultaneous TGA-DTA-DSC analyzer (TA Instruments) in an open platinum crucible under a dry argon atmosphere using approximately 10 mg of the sample (100 mL/min flow rate, from ambient temperature to 800 °C, 10 °C/min heating rate). Before analysis, all samples were dried to a constant mass at 80 °C under dry argon.

### Electron Paramagnetic Resonance (EPR) Spectroscopy

EPR spectra were recorded on an X-band Adani SPINSCAN X machine with the following parameters: modulation amplitude, 200 μT; sweep width, 12 μT; time constant, 38 ms; sweep time, 100 s; power, 0.285 mW.

## Supplementary Material



## Notes

Additional experimental details, supporting figures (TEM, SEM, Raman, XPS, DFT, TGA, kinetic equations, calculations), tables, and notes (DFT, theoretical calculations of the heating temperature, kinetic models, quantum yield calculations) (PDF)
