# Supplementary material for: Revising Model Reactions in Plasmonic Chemistry: From Nitrothiophenol Coupling to Alkoxyamine Homolysis
Source: ACS Catal. 2025 Jun 13;15(13):11163–76. doi: 10.1021/acscatal.5c01129 (PMC12235594; doi:10.1021/acscatal.5c01129)
Supplement: Supplementary file 1 [file cs5c01129_si_001.pdf]

Supporting information for

Revising Model Reactions in Plasmonic Chemistry: from Nitrothiophenol Coupling to Alkoxyamine Homolysis

Alina Gorbunova[a,b], Daria E. Votkina[a], Oleg Semyonov[a], Dmitry Kogolev[a], Jean-Patrick Joly[c], Sylvain R.A. Marque[c], Junais Habeeb Mokkah[d], Soniya Gahlawat[b], Markus Valtiner[b], Odile Chevalier[e], Pavel S. Postnikov[a,f], Olga Guselnikova[a, b *]

[a] *Research School of Chemistry and Applied Biomedical Sciences, Tomsk Polytechnic University, Tomsk, 634050, Russian Federation*

[b] *Institute of Applied Physics, Vienna University of Technology, 1040 Vienna, Austria*

[c] *Aix-Marseille University, CNRS, UMR 7273, ICR case 551, Avenue Escadrille Normandie-Niemen, Marseille 13397 Cedex 20, France*

[d] *College of Integrative Studies, Abdullah Al Salem University (AASU), Block 3, Khaldiya, Kuwait*

[e] *Aix-Marseille University, Avenue Escadrille Normandie-Niemen, Marseille 13397 Cedex 20, France*

[f] *Department of Solid-State Engineering, Institute of Chemical Technology, Prague 16628, Czech Republic*

* Correspondence to: olga.guselnikova@tuwien.ac.at, guselnikovaoa@tpu.ru

Table of content

Supplementary Note 1 - Preliminary test of X-Ray irradiation damage on the Au-S stability............5
Supplementary Note 2 – XPS database for N1s and S2p deconvolution..6
Supplementary Note 3 – XPS study of background signal at N1s..8
Supplementary Note 4 – Surface coverage calculations 9
Supplementary Note 5 – Monitoring of azo coupling by Raman spectroscopy................................10
Supplementary Note 6 – Raman spectroscopy for sulfur containing bond estimation 13
Supplementary Note 7 – Fractional life time method to determination of reaction order 17
Supplementary Note 8 – Assumptions for kinetic model based on XPS results...............................18
Supplementary Note 9 – Example of failed kinetic model 19
Supplementary Note 10 – Kinetic model calculations for suggested reaction pathways..................21
Supplementary Note 11 – Density functional theory (DFT) calculations...25
Supplementary Note 12 – Thermogravimetric analysis..26
Supplementary Note 13 – Theoretical calculations of heating temperature 27
Supplementary Note 14 – Temperature-dependent Raman spectroscopy...28
Supplementary Note 15 – Plasmon-driven C-ON bond homolysis of alkoxyamine at different wavelength LED irradiation. 29
Supplementary Note 16 – Quantum Yield calculation..30
Supplementary Note 17 – Plasmon-driven C-ON bond homolysis of alkoxyamine at different power LED irradiation...31
References 33

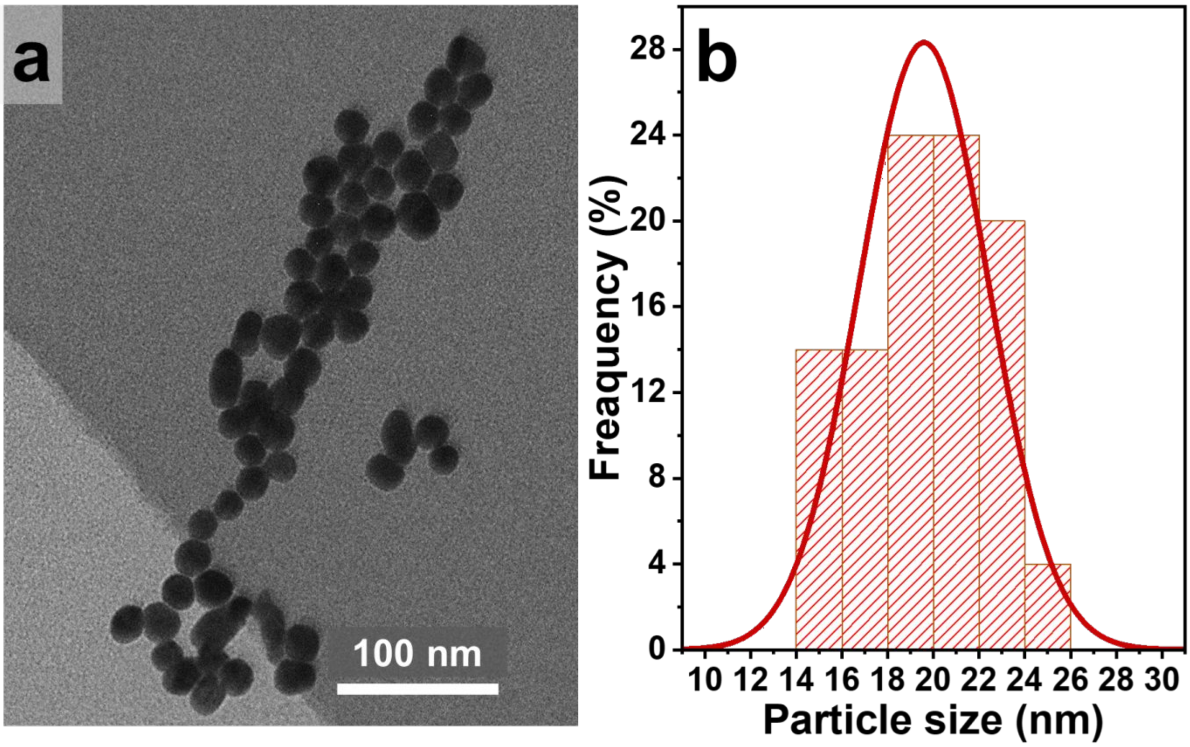

Figure S1. Characterization of Au NTP by electron microscopy: a) TEM image of Au NPs; b) size distribution according to TEM images.

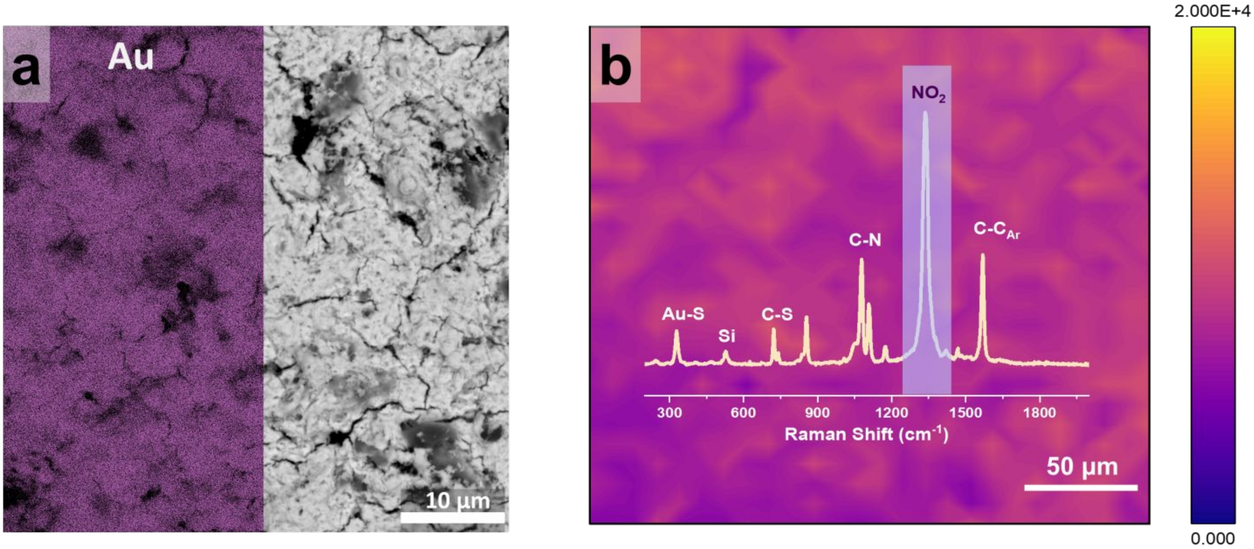

Figure S2. a) SEM image and EDX maps taken from prepared substrate; b) SERS mapping 1330 cm^{-1} peak intensity on Au-NTP (633 nm, 3 kW/cm^2) and Raman spectrum of Au-NTP

Table S1. Assignation of Raman peaks of Au-NTP and DMAB according to [3–5]

Raman Shift, cm^{-1}	Assignment
**Au-NTP on Si**	
330	Au-S
520	Si
720	C-S stretch
740	Ring breathing
846	C-H out of plane deformation
1074	C-H bending, C-S stretching, C-N stretching
1100	C-H bending, ring breathing
1175	CH bending
1330	NO_2 stretching
1410	Ring stretching
1469	C-H bending
1562	C-C stretching
2590	S-H stretching
**DMAB**	
1135	C-H bending; C-N stretching
1391	N=N stretching
1430	N=N stretching

Supplementary Note 1 - Preliminary test of X-Ray irradiation damage on the Au-S stability

To avoid influence of X-Ray irradiation on the Au-S stability additional optimization of XPS conditions was performed. Initial substrate Au-NTP was analyzed using different pass energies: 50, 60, 70, 80, 100 eV. Noticeably, oxidation processes were occurred at values above 70 eV, while desorption was observed above 80 eV (Figure S3). Therefore, for subsequent analysis pass energy 50 eV for high-resolution spectra recording was used.

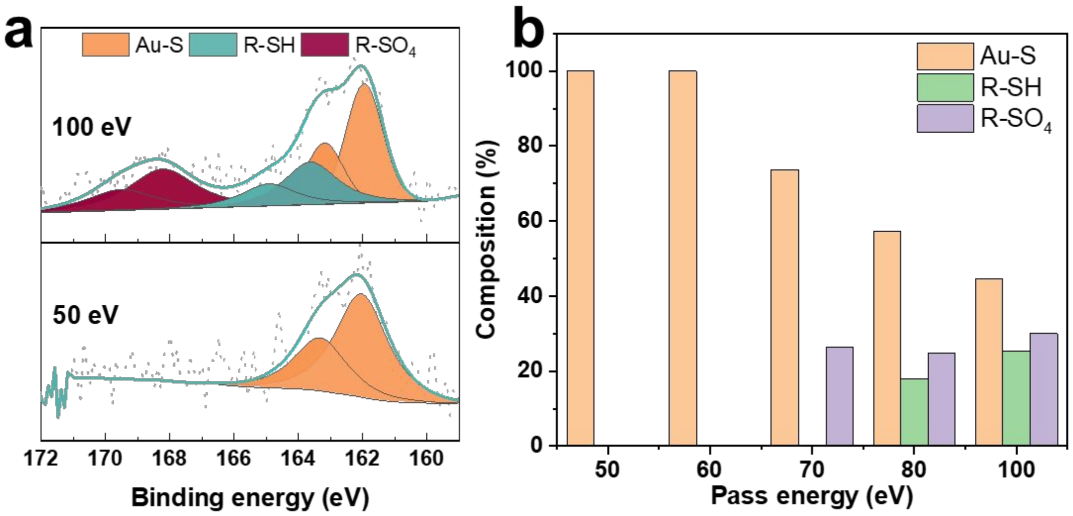

Figure S3. Influence X-Ray irradiation on the Au-S bond: a) High-resolution spectra (S2p) of Au-NTP at pass energy 50 and 100 eV; b) Composition of sulphur states of Au-NTP at different pass energies (50, 60, 70, 80, 100 eV)

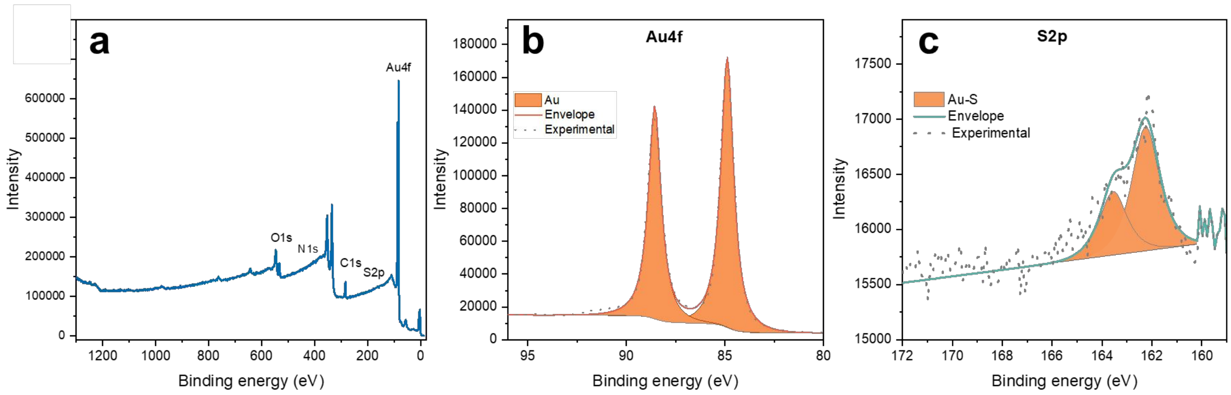

Figure S4. Characterization of Au-NTP surface by XPS: a) survey spectrum; b) high resolution of Au4f region; c) high resolution of S2p region.

Supplementary Note 2 – XPS database for N1s and S2p deconvolution

Additional experiments (Figure S5) were carried out to properly deconvolute high-resolution spectra in N1s and S2p regions. XPS of *p*-nitrothiophenol (PNTP) powder were measured to confirm presence of non-bonded thiol R-SH at 164 eV and nitro-group at 406 eV (Table S2, Figure S5c, d). Differences between nitro- (NO_2), amino- (NH_2) and diazo (-N=N-) groups were discovered by using *p*-nitroaniline (PNA) and *p*-aminoazobenzene (AAB) as references. According to XPS spectra (Figure S4 and Table S2), binding energy of NO_2 signal located at 406.4 eV, NH_2 at 399 eV, -N=N- at 400 eV respectively.

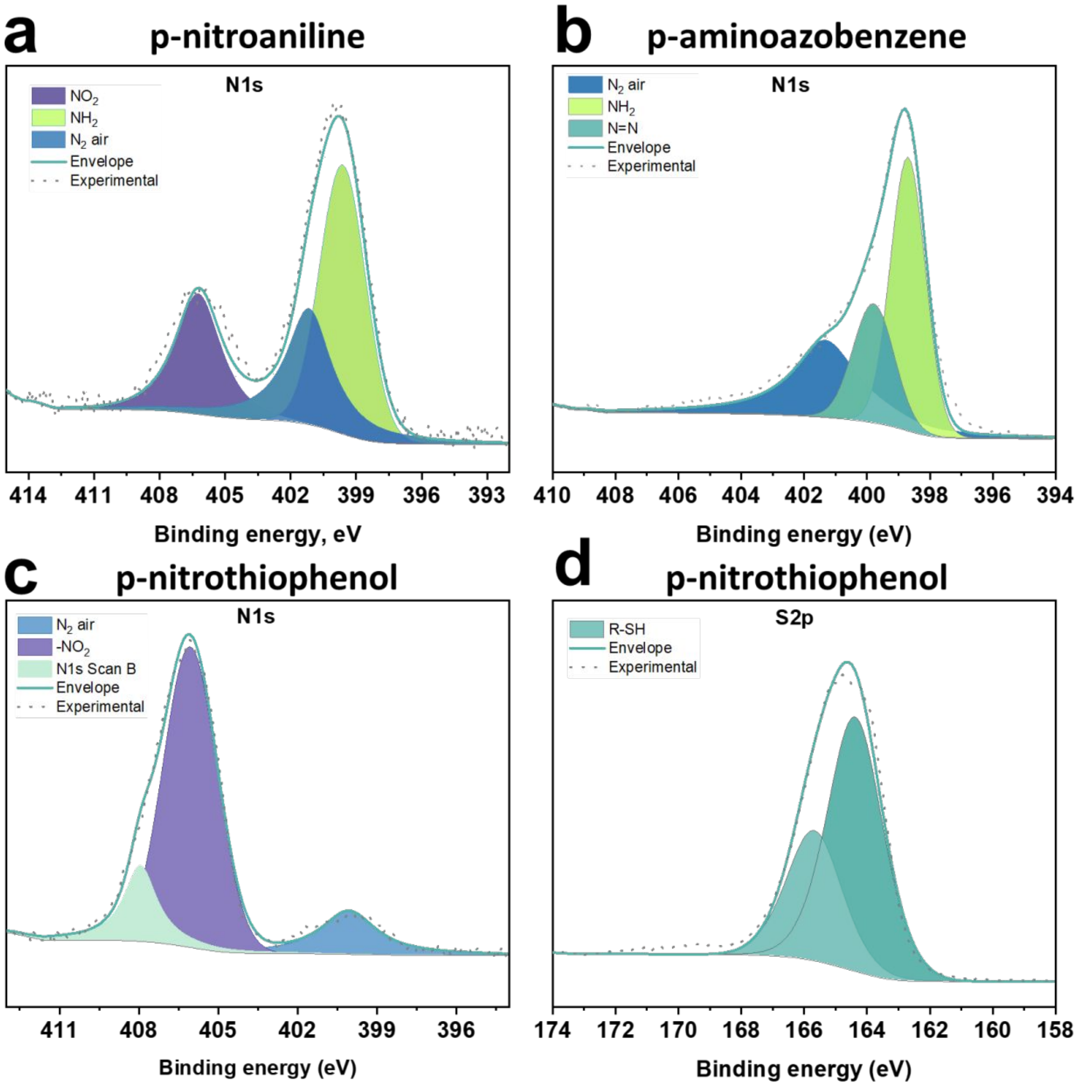

Figure S5. High-resolution spectra of references: a) N1s region of *p*-nitroaniline (PNA); b) N1s region of *p*-aminoazobenzene (AAB); c) N1s region of *p*-nitrothiophenol and d) S2p region of *p*-nitrothiophenol– unbonded thiols R-SH at 164 eV.

Table S2. Assignation of S2p and N1s chemical states at high-resolution XPS spectra according to experimental references (Figure S5) and literature.

Peak	Peak (BE)	FWHM (eV)	Experimental reference	Ref.
			**N1s**	
**NO_2**	406.4	2.15	PNTP, PNA	6
**N air**	400.9	2.40	AuNPs, PNTP	7,8
**NH_2**	398.6	2,46	PNA, AAB	6
**-N=N-**	400.2	1.54	AAB	9
			**S2p**	
**Au-S**	162.5	1.44	Au-NTP	10
**R-SH**	164.4	2.08	PNTP	10,11
**S_{ox}**	168.7	2.1	-	11

PNTP: *p*-nitrothiophenol; PNA: *p*-nitroaniline; AAB: 4-aminoazobenzene

Supplementary Note 3 – XPS study of background signal at N1s

The signal at 400 eV doesn't correspond to nitrogen states of prepared Au-NTP substrate. This signal can be related to contamination from air and nitrogen in transfer lines of XPS machines (high-purity N_2 (99.9999%) was used in transfer lines). This fact was reported previously elsewhere [7,8,12–15]. To determine the nature of the 400 eV signal, additional measurements were carried out on purified Au NPs deposited on Si, which were purged with argon and nitrogen. Figure S6 demonstrate high resolution spectra at N1s region for Au-NTP, Au NPs flushed by Ar and Au NPs flushed by N_2, where signal at 400 eV is observed. It is noteworthy that the signal area increases with slight shift of binding energy to 401 eV when the surface of the nanoparticles is purged with nitrogen. Thus, in further studies, the signal N_{air} in the region of 401 eV was taken into account during deconvolution.

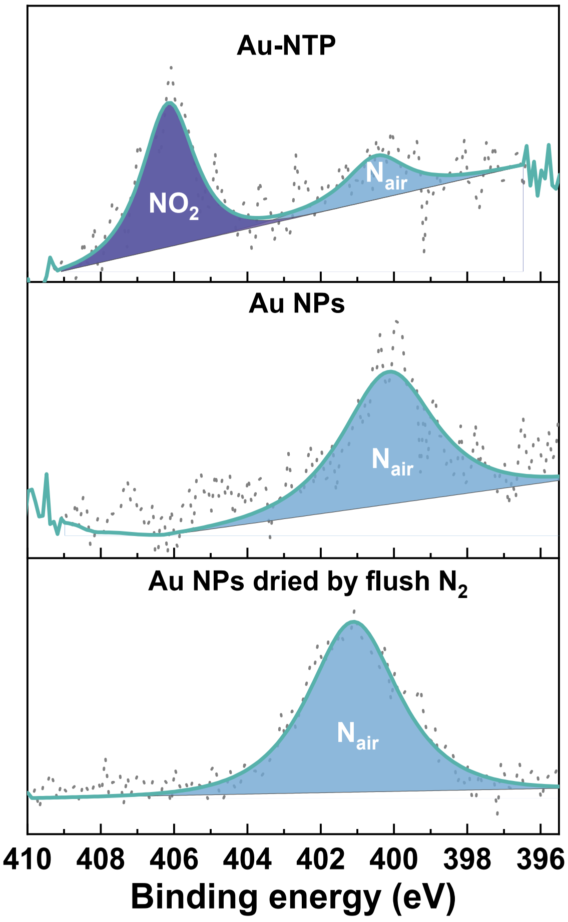

Figure S6. High resolution spectra N1s region presence of N_{air} signal at 401 eV for Au-NTP; Au NPs dried under flush of Ar and Au NPs dried under flush of N_2.

Supplementary Note 4 – Surface coverage calculations

The thickness of the p-nitrothiophenol layer on Au NPs can be evaluated based on the decrease in the Au4f XPS signal. The Au $4f_{7/2}$ peak was used for calculating the organic film thickness. The thickness of the organic film can be estimated from the surface chemical composition and signal intensities, according to ref. [16]. We observed gold signal (Au $4f_{7/2}$; 83.1–86.7 eV) attenuation after modification because functional groups were attached and the surface was covered; consequently, the organic layer thickness can be calculated using the following equation:

$$\frac{I}{I_0} = e^{-\frac{d}{\lambda \cdot sin\theta}} \quad \text{(eq. S1)}$$

where d – organic layer thickness;
λ – mean free path of the substrate-specific photoelectron in the organic layer;
θ – the analysis take-off angle relative to the surface (90° in the present study);
I/I_0 – ratio of the $Au4f_{7/2}$ peak intensities (before and after modification).

The value of λ was deduced from the empirical formula derived by Seah and Dench. [16]

$$\lambda_k = \frac{A_n}{E_k^2} + \frac{B_n}{E_k^{1/2}} \quad \text{(eq. S2)}$$

where E_k is the kinetic energy of photoelectrons.

For an Al Kα source, $E_k = 1486.6 - E_B$, where E_B is the binding energy of Au4f. If the substrate is coated with organic materials, $A_n = 49$ and $B_n = 0.11$, the unit of λ is $mg \cdot m^{-2}$, and the unit of energy is eV. To convert λ into nanometer units, λ in $mg \cdot m^{-2}$ must be divided by the density of the overlayers, which is assumed here to be equal to 1.0 $g \cdot cm^{-3}$. For $Au4f_{7/2}$, λ_k is calculated to be 4.12 $mg \cdot m^{-2}$, and the p-nitrothiophenol layer thickness is found to be 1.8 nm. The surface coverage for Au NPs grafted with PNTP was calculated according to eq. S3 (molar weight is 154 g/mol)[17]:

$$\Gamma = \frac{\rho \cdot d}{M} \quad \text{(eq. S3)}$$

Surface coverage of PNTP on Au NPs is amounted to 9.5 mol/nm^2.

Supplementary Note 5 – Monitoring of azo coupling by Raman spectroscopy

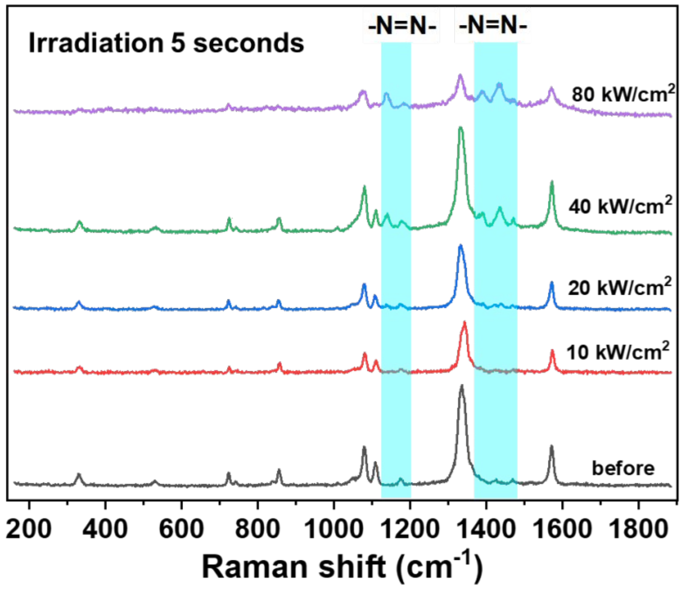

Figure S7. Raman spectra of Au-NTP: after 5 second irradiation at power density 10-80 kW/cm^2.

Table S3. Overview of experimental parameters in previously published papers

№	Wavelength, nm	Irradiance	Photons number	Time, s	Ref.
1	514	94 kW/cm^2	$1.3 \cdot 10^{17}$	98	[18]
2	785	0.5 kW/cm^2	$1.8 \cdot 10^{17}$	30	[19]
3	785	0.9 kW/cm^2	$2.3 \cdot 10^{19}$	600	[20]
4	633	0,8-17 kW/cm^2	$1.4 \cdot 10^{10}$	50	[21]
5	633	313 kW/cm^2	$3.1 \cdot 10^{12}$	700	[22]
6	633	4000 kW/cm^2	$1.9 \cdot 10^{25}$	30	[23]
7	633	83.5 kW/cm^2	$7 \cdot 10^{9}$	22	[24]
8	**633**	**80 kW/cm^2**	**$8.0 \cdot 10^{16}$**	**5**	**This work**
9	**633**	**22 kW/cm^2**	**$2.2 \cdot 10^{16}$**	**60**	**This work**

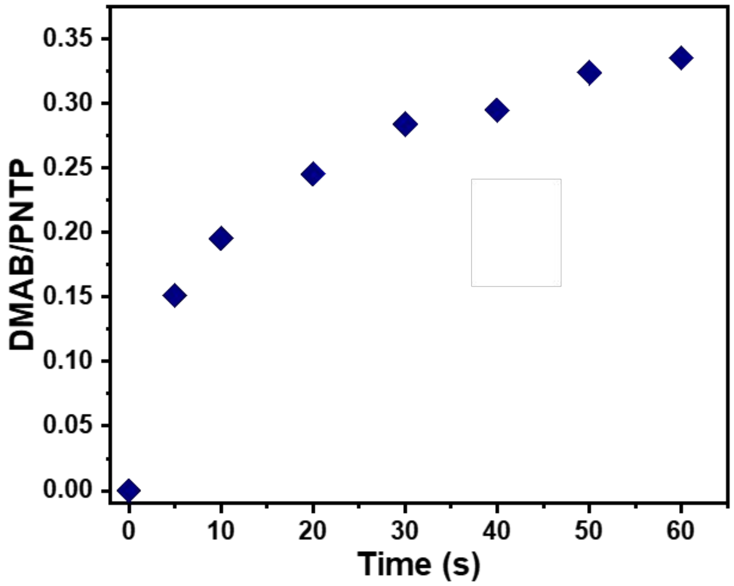

Figure S8. Changes in DMAB/PNTP (1430/1330 cm^{-1}) intensities during plasmon-driven PNTP azo coupling under irradiation with power density 22 kW/cm^2 over 60 seconds

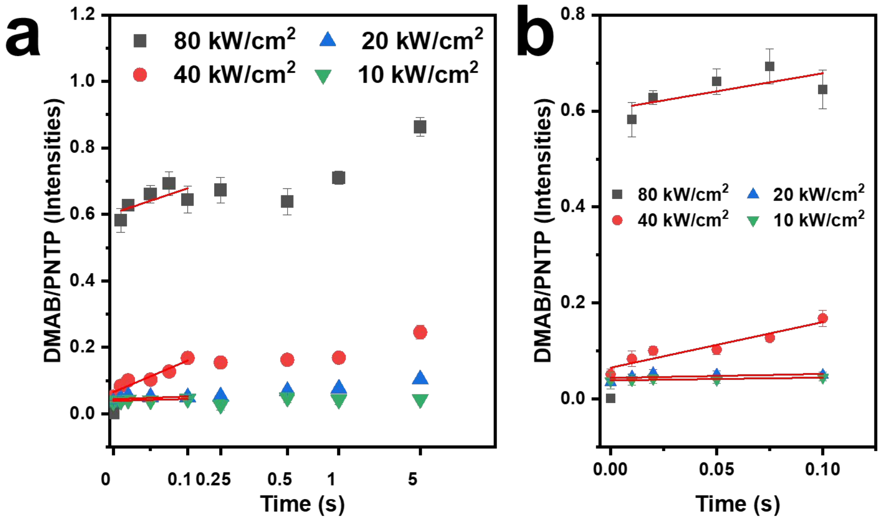

Figure S9. a) Changes in DMAB/PNTP (1430/1330 cm^{-1}) intensities during plasmon-driven PNTP azo coupling under irradiation with power density 10, 20, 40, 80 kW/cm^2 over 5 seconds; b) Fitting of initial linear region in 0 – 0.1 sec interval (red lines represent the initial slope linear fitting)

Table S4. Evaluation of kinetic parameters of azo coupling by Raman spectroscopy.

**Power, kW/cm^2**	**1st process**	
	**Initial slope rate^a, s^{-1}**	**R^{2b}**

**10**	0.06	0.73
**20**	0.09	0.48
**40**	0.95	0.9
**80**	0.75	0.47

[a] Initial slope rate was obtained from linear fitting of kinetic curves in 0 – 0.1 s time interval.

[b] R^2 was calculated from fitting using equation $R^2 = 1 - \frac{\sum(y_i - \hat{y}_i)^2}{\sum(y_i - \bar{y})^2}$

The ratio of DMAB/PNTP intensities (1430/1330 cm^{-1}) was used to reveal changes in the concentration of surface groups (Figure S7) similar to previously published reports (summarized in Table S3) [18,20,22,25]. Further approximation of data with specific function has to provides the rate constants to estimate the relation between power irradiation and reaction efficacy (Figure S9). However, the fitting of observed curves with 1st, 2nd and 3rd order did not give high enough R^2. We revealed that the initial (0 – 0.1 s) linear region could be fitted with high R^2 values (Figure S9, Table S4). The reaction rates were found to be 0.06 s^{-1} for 10 kW/cm^2, 0.09 s^{-1} for 20 kW/cm^2, and 0.95 s^{-1} for 40 kW/cm^2 and 0.75 s^{-1} for 80 kW/cm^2 (Table S4). After the first second, the N=N related signal still slowly increases reaching the plateau values.

Supplementary Note 6 – Raman spectroscopy for sulfur containing bond estimation

To estimate the structural changes in sulfur-containing groups under PNTP dimerization at 80 kW/cm^2, Raman spectra were collected at the range 250-2800 cm^{-1} with laser power density at 3 kW/cm^2 (1 second, 1 scan). According to Figure S10, there are three distinct weak signals related to sulfur contained bonds appear, specifically Au-S at 330 cm^{-1}, C-S at 730 cm^{-1} and S-H at 2645 cm^{-1}.[26–28] Moreover, Raman shift for S-O bond (1030-1100 cm^{-1})[28] can be overlapped by other signals from PNTP, such as C-N stretching vibrations at 1074 cm^{-1}. Despite presence of Au-S, C-S and S-H bonds at the Raman spectra it is impossible to conclude about possible processes due to its weak intensity. To the best of our knowledge, commonly, Raman spectra are considered with range 1000-1800 cm^{-1} using only N=N and NO_2 signals for studying PNTP/PATP dimerization.[22,25]

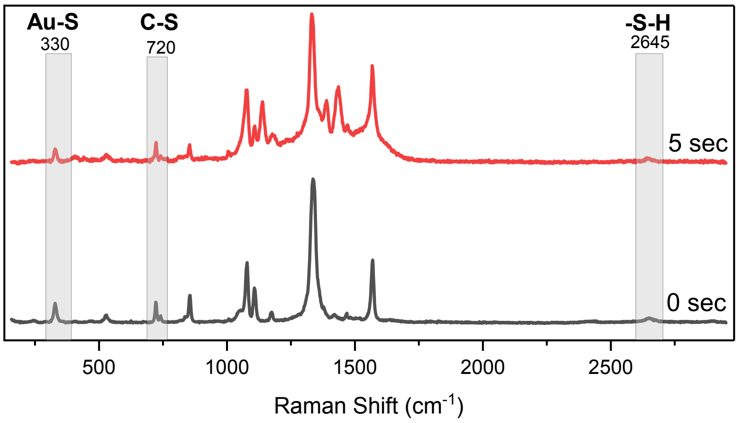

Figure S10. Raman spectra in 160 – 2900 cm^{-1} region of Au-NTP before and after irradiation with laser power density 80 kW/cm^2 containing Au-S, C-S and S-H signals

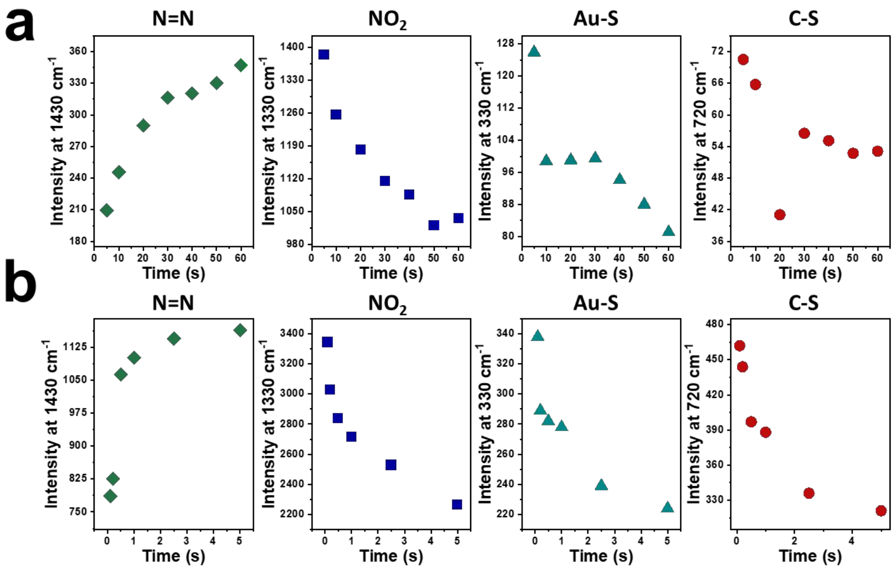

Figure S11. Raman intensity spectra changes (N=N, NO_2, Au) during plasmon driven azo coupling at a) 22 kW/cm^2 in 0-60 seconds range b) 80 kW/cm^2 in 0-5 second range

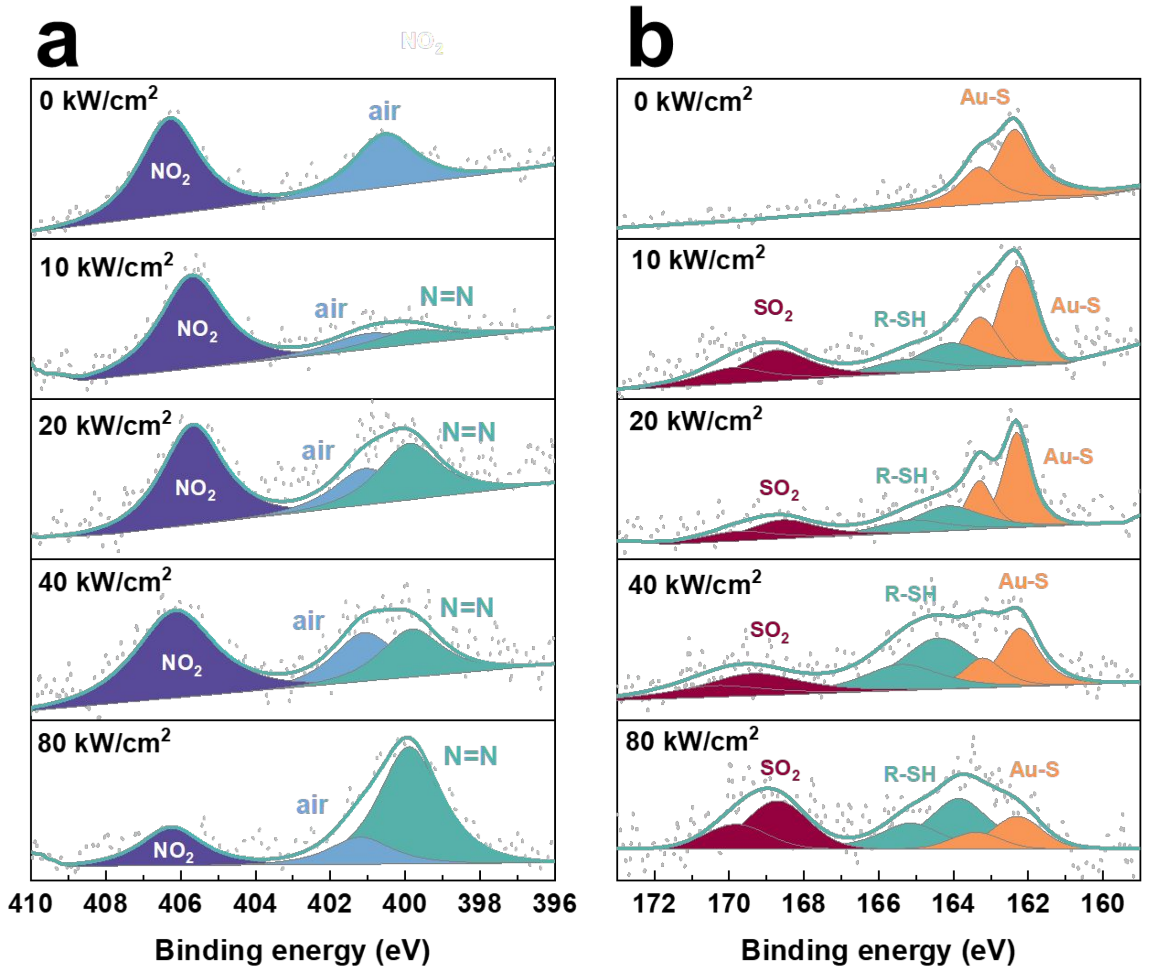

Figure S12. High resolution spectra after azo coupling under different power density (0, 10, 20, 40, 80 kW/cm^2) at a) N1s region and b) S2p region.

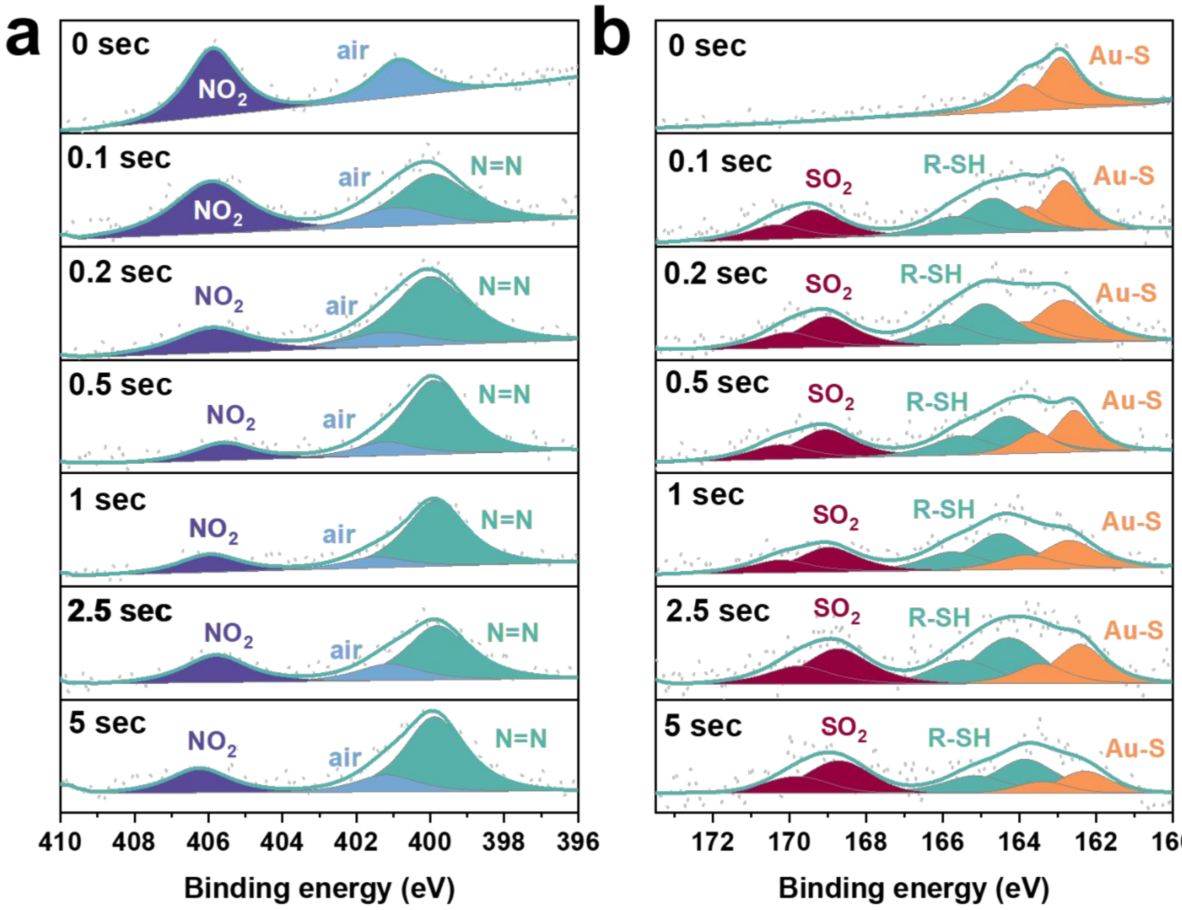

Figure S13. High resolution XPS spectra after azo coupling under 80 kW/cm^2 with different irradiation time (0, 0.1, 0.2, 0.5, 1, 2.5, 5 seconds) at: a) N1s region and b) S2p region.

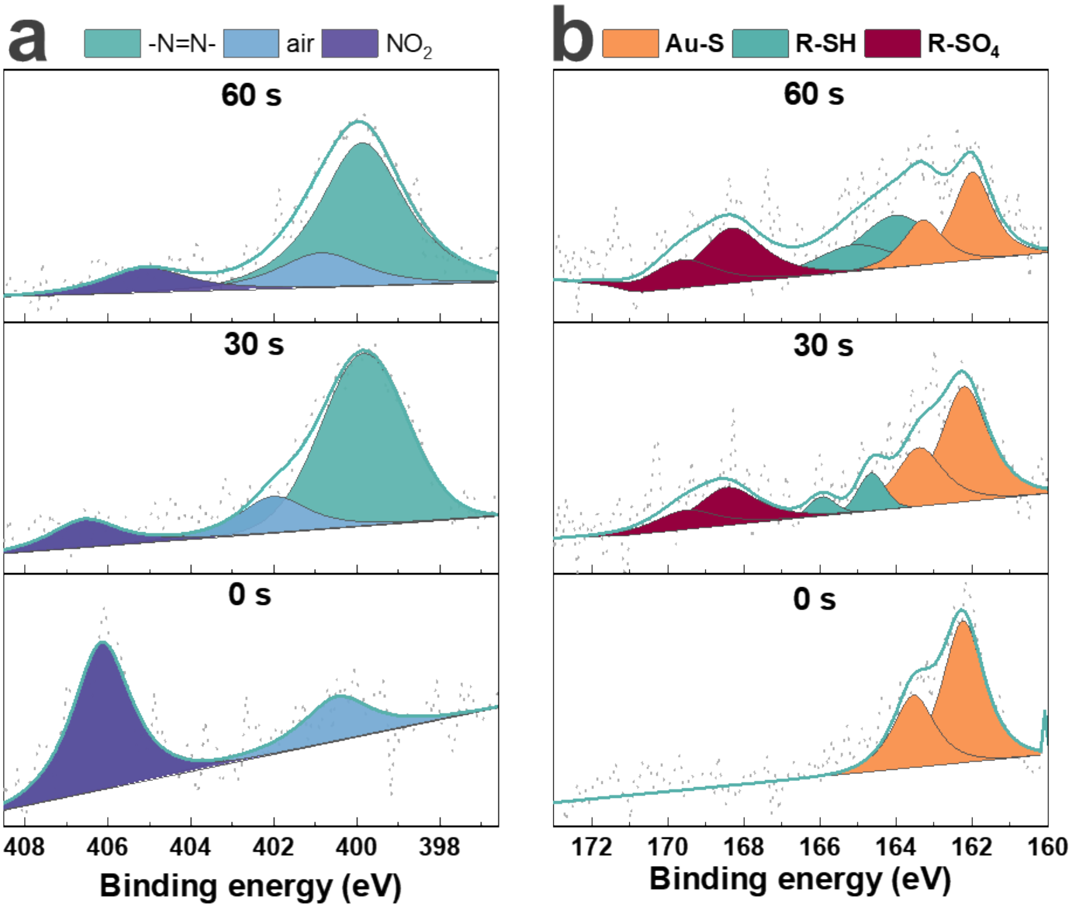

Figure S14. High resolution spectra after azo coupling at 22 kW/cm^2 (0, 30, 60 seconds) at a) N1s region and b) S2p region.

Supplementary Note 7 – Fractional life time method to determination of reaction order

General reaction order was attempted to be calculated by fractional lifetime method. The specified fractional life (*e.g.* half-life) is determined for a number of different concentrations that depend on order (if n≠1) by equation:

$$\frac{t_{1/q}}{t_{1/p}} = \frac{\left(1-\frac{1}{q}\right)^{1-n}-1}{\left(1-\frac{1}{p}\right)^{1-n}-1} \quad \text{(eq. S4)},$$

Where n is reaction order; q, p – conversion C_{n1}/C_0 and C_{n2}/C_0; $t_{1/q}$, $t_{1/p}$ – fractional life time at q and p, respectively. In case of first order n =1 equation is:

$$\frac{t_{\frac{1}{q}}}{t_{\frac{1}{p}}} = \frac{\ln\left(1-\frac{1}{q}\right)}{\ln\left(1-\frac{1}{q}\right)} \quad \text{(eq. S5)},$$

According to the equations S4 and S5, curves were constructed depending on the ratio of fractional life times $\frac{t_{1/q}}{t_{1/p}}$ on the reaction order, presented in the Figure S15. Then, based on experimental data, the values of $\frac{t_{1/q}}{t_{1/p}}$ were found for 1/q =1/3; 1/4; 1/2; 2/3; 3/4 and 1/p = 1/3; 1/4; 1/2; 2/3; 3/4, which are shown on the graph as star points (blue for Au-S and red for NO_2). The same order value should be observed on each plotted curve at different time ratios for determination of general reaction order. In our case **<u>we didn't observe general reaction order</u>.**

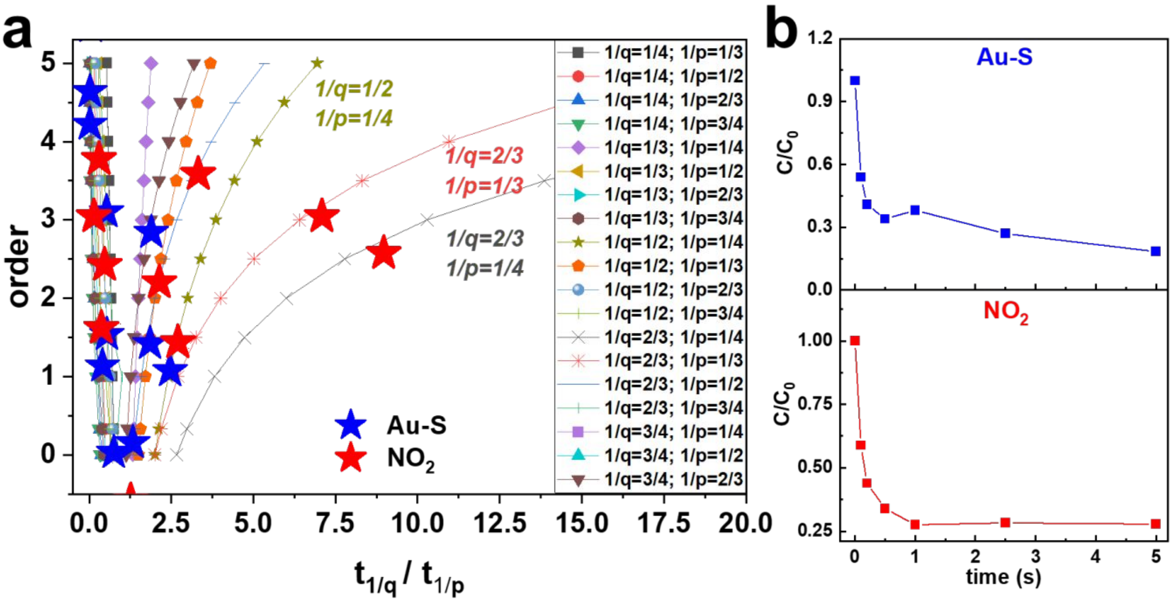

Figure S15. Fractional life time method: a) order reaction dependence from fractional life time in comparison with experimental data (blue star for Au-S and red star for NO_2); b) Experimental curves of Au-S and NO_2 conversion.

Supplementary Note 8 – Assumptions for kinetic model based on XPS results

(i) Reversibility

The cleavage of Au—S bond is assumed to be reversible. Thus, it means that the back reaction of **A→B**, **G→C**, **C→D** and **E→F** should be included in the kinetic schemes. However, the back-reaction **C → G** is disregarded because **C** is likely to be the *trans*- isomer implying an isomerization to the form *cis*- which is not thermodynamically favored. Moreover, the back-reaction **D → C** and **F → E** (and **F→** detached SO_x oxidized PNTP) are disregarded because of the steric hindrance due to the bulkiness of the molecules (roughly twice the size of **A**). On the other hand, the back-reaction **B → A** is included as it regenerates the main reactive **A** and it will be assumed an order 0^{ed} or 1st. Thus, the reaction **A→B**, **G→C**, **C→D** and **E→F**, which are all Au-S bond cleavage events, should display the same order, i.e., 0^{ed} or 1st. For reaction **B→X**, only non-reversible reactions are assumed and for the sake of simplicity and of clarity only 1st order is assumed.

(ii) Excess of one of reagent

A 2nd order reaction is not assumed for the reaction **B → A** because the surface is very large compared to the molecule **B** and then a 0^{ed} or a pseudo-1st order reaction is more likely. Obviously, reactions **B→C**, **A→G**, and **C→E** are 2nd order reactions. However, reactant **A** is expected to be in large excess regarding species **B** ([**A**]>>[**B**]) meaning that the 2nd order reaction is transformed into pseudo-1st order reaction or 0^{ed} order. For the reaction **C→E**, O_2 is assumed also to be in large excess meaning a pseudo-1st order reaction instead of a 2nd order reaction.

(iii) Multi-step processes

As the reaction **A→G** is a multi-step process, simple orders 0^{ed}, 1st and 2nd are assumed. Because the reaction **C→E** is also a multi-step process, 0^{ed} and pseudo-1st order are assumed. For reaction **B→X**, only non-reversible reactions of 1st order are assumed without explicitly modeling oxidation, as any oxidized **B** species would rapidly re-adsorbed to **A** or involved in further coupling reactions producing intermediates **C, D,** and **E**. For reaction **B→X**, only non-reversible reactions are assumed and for the sake of simplicity and of clarity only 1st order is assumed.

Thus, assuming a first order for the reactions involving it leaves us with 4 reactions **A→G** assuming order 0^{ed}, 1st and 2nd, and orders 0^{ed} and1^{st} for **B→A**, **B→C**, **C→E**.

Supplementary Note 9 – Example of failed kinetic model

The kinetic equations are developed for what was looking as the most likely model:

Reaction	1	2	3	4	5	6	7	8
	A→G	A→B	B→X	B→C	C→D	C→E	G→C	E→F
Order	2	1	1	1	1	0	1	1

B is considered as an intermediate and approximation of quasi-steady states (AEQS) is applied

$$\frac{d[B]}{dt} = 0 = k_2[A] - k_{-2}[B] - k_3[B] - k_4[B] \quad \text{(eq. S6)}$$

$$[B] = \frac{k_2}{k_{-2}+k_3+k_4}[A] = \alpha_1[A] \quad \text{(eq. S7)}$$

$$-\frac{d[A]}{dt} = k_2[A] - k_{-2}[B] + k_4[B] + k_1[A]^2 = k_2[A] - k_{-2}\alpha_1[A] + k_4\alpha_1[A] + k_1[A]^2 = k_1[A]^2 + (k_2 - k_{-2}\alpha_1 + k_4\alpha_1)[A] = k_1[A]^2 + \alpha_2[A] \quad \text{(eq. S8)}$$

$$[NO_2] = [A] = \frac{\alpha_2 e^{(-\alpha_2 t + c)}}{1 - k_1 e^{(-\alpha_2 t + c)}} \quad \text{(eq. S9)}$$

At t=∞, $[A]_\infty$=0, involving α_2>0, in always time is

$$\alpha_2 = k_2 - k_{-2}\alpha_1 + k_4\alpha_1 > 0 \quad \text{(eq. S10)}$$

$$\alpha_2 = \frac{k_2\ (2k_4+k_3)}{k_3+k_4+k_{-2}} \quad \text{(eq. S11)}$$

Keeping in mind that k_i is always positive. Hence, **C** is given as eq. S12 at t=0

$$C = \ln\left(\frac{[A]_0}{\alpha_2+[A]_0 k_1}\right) \quad \text{(eq. S12)}$$

$$\frac{d[G]}{dt} = k_1[A]^2 - k_7[G] \quad \text{(eq. S13)}$$

$$=> \frac{d[G]}{dt} + k_7[G] = k_1\left(\frac{\alpha_2 e^{(-\alpha_2 t + c)}}{1 - k_1 e^{(-\alpha_2 t + c)}}\right)^2 \quad \text{(eq. S14)}$$

$$\frac{d[C]}{dt} = k_4[B] + k_7[G] - k_5[C] - k_6 \quad \text{(eq. S15)}$$

To solve this differential equation, it is assumed that the numerical solutions of species **G** is negligible to other terms. Then, it affords:

$$\frac{d[C]}{dt} = k_4\alpha_1[A] - k_5[C] - k_6 \quad \text{(eq. S16)}$$

$$\frac{d[C]}{dt} + k_5[C] = k_4\alpha_1\frac{\alpha_2 e^{(-\alpha_2 t + c)}}{1 - k_1 e^{(-\alpha_2 t + c)}} - k_6 = \frac{\alpha_3 e^{(-\alpha_2 t + c)}}{1 - k_1 e^{(-\alpha_2 t + c)}} - k_6 \quad \text{(eq. S17)}$$

$$\frac{d[E]}{dt} = k_6 - k_8[E] \quad \text{(eq. S18)}$$

$$\frac{d[E]}{dt} + k_8[E] = k_6 \quad \text{(eq. S19)}$$

$$(dE + k_8[E]dt)e^{k_8 t} = k_6 e^{k_8 t} \quad \text{(eq. S20)}$$

$$\int_0^t [E]e^{k_8 t} = \frac{k_6}{k_8}\int_0^t e^{k_8 t} \quad \text{(eq. S21)}$$

$$[E]e^{k_8 t} = \frac{k_6}{k_8}(e^{k_8 t} - 1) \quad \text{(eq. S22)}$$

$$[E] = \frac{k_6}{k_8}(1 - e^{-k_8 t}) \quad \text{(eq. S23)}$$

$$\frac{d[N{=}N]}{dt} = k_1[A]^2 + k_7[G] + k_4[B] - k_5[C] - k_6 = k_4\alpha_1[A] + k_1[A]^2 - k_6 + k_7[G] - k_5[C] = k_4 \alpha_1 \frac{\alpha_2 e^{(-\alpha_2 t + c)}}{1 - k_1 e^{(-\alpha_2 t + c)}} + k_1\left(\frac{\alpha_2 e^{(-\alpha_2 t + c)}}{1 - k_1 e^{(-\alpha_2 t + c)}}\right)^2 - k_6 + k_7[G] - k_5[C] \quad \text{(eq. S24)}$$

$$\frac{d[SH]}{dt} = \frac{d[E]}{dt} = k_6 - k_8[E] \quad \text{(eq. S25)}$$

$$\frac{d[E]}{dt} + k_8[E] = k_6 \Rightarrow [SO_2] = [E] = \frac{k_6}{k_8}(1 - e^{-k_8 t}) \quad \text{(eq. S26)}$$

$$\frac{d[SH]}{dt} = k_4[B] + k_7[G] - k_5[C] - k_6 = k_4\alpha_1 \frac{\alpha_2 e^{(-\alpha_2 t + c)}}{1 - k_1 e^{(-\alpha_2 t + c)}} + k_7[G] - k_5[C] - k_6 = \frac{\alpha_5 e^{(-\alpha_2 t + c)}}{1 - k_1 e^{(-\alpha_2 t + c)}} + k_7[G] - k_5[C] - k_6 \quad \text{(eq. S27)}$$

$$-\frac{d[Au{-}S]}{dt} = k_2[A] - k_{-2}[B] + k_7[G] + k_5[C] + k_8[E] = k_2[A] - k_{-2}\alpha_1[A] + k_8\frac{k_6}{k_8}(1 - e^{-k_8 t}) + k_7[G] + k_5[C] = k_2 \frac{\alpha_2 e^{(-\alpha_2 t + c)}}{1 - k_1 e^{(-\alpha_2 t + c)}} - k_{-2}\alpha_1 \frac{\alpha_2 e^{(-\alpha_2 t + c)}}{1 - k_1 e^{(-\alpha_2 t + c)}} + k_6(1 - e^{-k_8 t}) + k_7[G] + k_5[C] = \alpha_2 (k_2 - k_{-2}\alpha_1) \frac{e^{(-\alpha_2 t + c)}}{1 - k_1 e^{(-\alpha_2 t + c)}} + k_6(1 - e^{-k_8 t}) + k_7[G] + k_5[C] = \alpha_6 \frac{e^{(-\alpha_2 t + c)}}{1 - k_1 e^{(-\alpha_2 t + c)}} + k_6(1 - e^{-k_8 t}) + k_7[G] + k_5[C] \quad \text{(eq. S28)}$$

Obtained kinetic model for $[NO_2]$ as eq. S9 was fitted with experimental data (Figure S16). Despite R^2=0.96 α_2 is negative and has no physical sense according eq. S10 and S11.

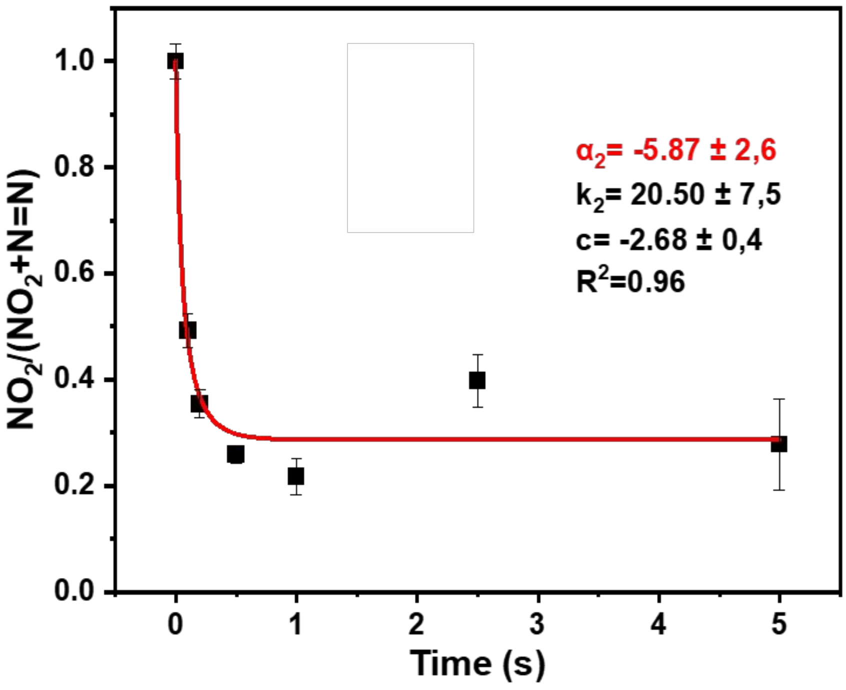

Figure S16. Kinetic curve tracked by changes in NO_2/(N=N+NO2) at 80 kW/cm^2 with fitting using eq. S9

Supplementary Note 10 – Kinetic model calculations for suggested reaction pathways

The kinetic equations are developed for what was looking as the most likely model:

Reaction	1	2	3	4	5	6	7	8
	A→G	A→B	B→X	B→C	C→D	C→E	G→C	E→F
Order	0	1	1	1	1	0	1	1

According XPS results and suggested reactions $[NO_2] = [A]$; $[SH] = [E]$; $[SO_2] = [G]$;

$$-\frac{dA}{dt} = k_2[A] - k_{-2} + k_4[B] + k_1 = k_2[A] - k_{-2} + k_4\alpha_1[A] - k_4\alpha_2 + k_1 \quad \text{(eq. S29)}$$

$$-\frac{dA}{dt} = (k_2 + k_4\alpha_1)[A] + (k_1 - k_{-2} - k_4\alpha_2) = \alpha_3[A] + \alpha_4 \quad \text{(eq. S30)}$$

where $\alpha_3 = k_2 + k_4\alpha_1$ (eq. S31) and $\alpha_4 = k_1 - k_{-2} - k_4\alpha_2$(eq. S32)

$$-\frac{dA}{dt} - \alpha_3[A] = \alpha_4 \quad \text{(eq. S33)}$$

$$\frac{dA}{dt} + \alpha_3[A] = -\alpha_4 \quad \text{(eq. S34)}$$

$$\int_0^t [A]e^{\alpha_3 t} = -\frac{\alpha_4}{\alpha_3}\int_0^t e^{\alpha_3 t} \quad \text{(eq. S35)}$$

$$[A]e^{\alpha_3 t} - [A]_0 = -\frac{\alpha_4}{\alpha_3}(e^{\alpha_3 t} - 1) \quad \text{(eq. S36)}$$

$$[A] = \left([A]_0 + \frac{\alpha_4}{\alpha_3}\right)e^{\alpha_3 t} - \frac{\alpha_4}{\alpha_3} \quad \text{(eq. S37)}$$

$$-\frac{dB}{dt} = k_2[A] - k_{-2} - k_3[B] - k_4[B] = 0 \quad \text{(eq. S38)}$$

$$[B] = \frac{k_2}{k_3+k_4}[A] - \frac{k_{-2}}{k_3+k_4} = \alpha_1[A] - \alpha_2 \quad \text{(eq. S39)}$$

where $\alpha_1 = \frac{k_2}{k_3+k_4}$ (eq. S40), $\alpha_2 = \frac{k_{-2}}{k_3+k_4}$(eq. S41);

when t→∞, $[A]_\infty = -\frac{\alpha_4}{\alpha_3} > 0$ (eq. S42)

$$\alpha_3 = k_2 + k_4\alpha_1 = k_2 + \frac{k_4 k_2}{k_3+k_4} > 0 \quad \text{(eq. S43)}$$

$$\alpha_4 = k_1 - k_{-2} - k_4\alpha_2 = k_1 - k_{-2} - \frac{k_4 k_{-2}}{k_3+k_4} < 0 \quad \text{(eq. S44)}$$

$$k_1 < k_2\left(\frac{2k_4+k_3}{k_3+k_4}\right) \quad \text{(eq. S45)}$$

$$-\frac{d[G]}{dt} = k_7[G] - k_1 \quad \text{(eq. S46)}$$

$$[G] = \frac{k_1}{k_7}(1 - e^{-k_7 t}) = \alpha_5(1 - e^{-k_7 t}) \quad \text{(eq. S47)}$$

where $\alpha_5 = \frac{k_1}{k_7}$(eq. S48);

$$\frac{d[E]}{dt} = k_6 - k_8[E] \quad \text{(eq. S49)}$$

$$[E] = \frac{k_6}{k_8}(1 - e^{-k_8 t}) = \alpha_6(1 - e^{-k_8 t}) \quad \text{(eq. S50)}$$

where $\alpha_6 = \frac{k_6}{k_8}$ (eq. S51);

$$\frac{d[C]}{dt} = k_4[B] + k_7[G] - k_5[C] - k_6 = k_4\alpha_1[A] - k_4\alpha_2 + k_7[G] - k_5[C] - k_6 \quad \text{(eq. S52)}$$

$$\frac{d[C]}{dt} + k_5[C] = k_4\alpha_1\left([A]_0 + \frac{\alpha_4}{\alpha_3}\right)e^{-\alpha_3 t} - k_4\frac{\alpha_1\alpha_4}{\alpha_3} - k_4\alpha_2 + k_7\alpha_5 - k_7\alpha_5 e^{-k_7 t} - k_6 \quad \text{(eq. S53)}$$

$$\frac{d[C]}{dt} + k_5[C] = \alpha_7 e^{-\alpha_3 t} - k_7\alpha_5 e^{-k_7 t} + \ \alpha_8 \quad \text{(eq. S54)}$$

where $\alpha_7 = k_4\alpha_1\left([A]_0 + \frac{\alpha_4}{\alpha_3}\right)$ (eq. S55), $\alpha_8 = k_7\alpha_5 - k_4\frac{\alpha_1\alpha_4}{\alpha_3} - k_4\alpha_2 - k_6$ (eq. S56)

$$[C]e^{k_5 t} = \frac{\alpha_7}{k_5 - \alpha_3}(e^{(k_5 - \alpha_3)t} - 1) - \frac{k_7\alpha_5}{k_5 - k_7}(e^{(k_5 - k_7)t} - 1) + \frac{\alpha_8}{k_5} \quad \text{(eq. S57)}$$

where $\alpha_9 = \frac{\alpha_7}{k_5 - \alpha_3}$ (eq. S58), $\alpha_{10} = \frac{k_7\alpha_5}{k_5 - k_7}$(eq. S59), $\alpha_{11} = \frac{\alpha_8}{k_5}$(eq. S60)

$$[C] = \alpha_9(e^{-\alpha_3 t} - e^{-k_5 t}) - \alpha_{10}(e^{-k_7 t} - e^{-k_5 t}) + \alpha_{11}(1 - e^{-k_5 t}) \quad \text{(eq. S61)}$$

$$[C] = (\alpha_{10} - \alpha_9 - \alpha_{11})e^{-k_5 t} + \alpha_9 e^{-\alpha_3 t} - \alpha_{10}e^{-k_7 t} + \alpha_{11} \quad \text{(eq. S62)}$$

where $\alpha_{11} \geq 0$, $k_6 \leq k_1(k_3 + k_4)$, $k_6 \leq k_{-2}$

$$[C] = \alpha_{12}e^{-k_5 t} + \alpha_9 e^{-\alpha_3 t} - \alpha_{10}e^{-k_7 t} + \alpha_{11} \quad \text{(eq. S63)}$$

where $\alpha_{12} = \alpha_{10} - \alpha_9 - \alpha_{11}$(eq. S64);

$$[N=N]=[G]+[C]+[E] \quad \text{(eq. S65)}$$

$$\frac{d[N=N]}{dt}=\frac{d[G]}{dt}+\frac{d[C]}{dt}+\frac{d[E]}{dt}=V_1-V_7+V_4-V_7-V_5-V_6+V_6-V_8=V_1+V_4-V_5-V_8 \quad \text{(eq. S66)}$$

$$[N=N]=[G]+[C]+[E]=\alpha_5-\alpha_5e^{-k_7t}+\alpha_{12}e^{-k_5t}+\alpha_9e^{-\alpha_3t}-\alpha_{10}e^{-k_7t}+\alpha_{11}+\alpha_6-\alpha_6e^{-k_8t}=\alpha_{12}e^{-k_5t}+\alpha_9e^{-\alpha_3t}-(\alpha_5+\alpha_{10})e^{-k_7t}-\alpha_6e^{-k_8t}+\alpha_5+\alpha_{11}+\alpha_6=\alpha_{12}e^{-k_5t}+\alpha_9e^{-\alpha_3t}-\alpha_{13}e^{-k_7t}-\alpha_6e^{-k_8t}+\alpha_{14} \quad \text{(eq. S67)}$$

where $\alpha_{13}=\alpha_5+\alpha_{10}$ (eq. S68) $\alpha_{14}=\alpha_5+\alpha_{11}+\alpha_6$

$$t\rightarrow\infty \Rightarrow [N=N]_\infty=\alpha_{14}>0$$

where $\alpha_{11}=\frac{\alpha_8}{k_5}=\frac{k_7\alpha_5}{k_5}-\frac{k_6}{k_5}-\frac{k_4\alpha_2}{k_5}-\frac{k_4\alpha_1\alpha_4}{k_5\alpha_3}$ (eq. S69) and $\alpha_{14}=\alpha_5+\alpha_6+\alpha_{11}=\frac{k_1}{k_7}+\frac{k_6}{k_8}+\frac{k_1}{k_5}-\frac{k_6}{k_5}-\frac{k_4k_{-2}}{k_5(k_3+k_4)}-\frac{k_4k_1}{k_5(k_3+2k_4)}+\frac{k_4k_{-2}}{k_5(k_3+k_4)}=\frac{k_1}{k_5k_7}(k_5+k_7)+\frac{k_6}{k_5k_8}(k_5-k_8)-\frac{k_4k_1}{k_5(k_3+2k_4)}>0$(eq. S70)

$$\Rightarrow \quad k_1>\frac{k_6k_7(k_3+2k_4)(k_8-k_5)}{k_8(k_5(k_3+2k_4)+k_7(k_3+k_4))} \quad \text{(eq. S71)}$$

where $\alpha_3=k_2+k_4\alpha_1$(eq. S31), $\alpha_3=\frac{k_2k_3+2k_4k_2}{k_3+k_4}$(eq. S72)

$$\alpha_4=k_1-k_{-2}-k_4\alpha_2=k_1-k_{-2}-\frac{k_4k_{-2}}{k_3+k_4}=\frac{(k_1-k_{-2})(k_3+k_4)-k_4k_{-2}}{k_3+k_4}=\frac{k_1(k_3+k_4)-k_{-2}k_3-2k_4k_{-2}}{k_3+k_4} \quad \frac{\alpha_4}{\alpha_3}=\frac{k_1(k_3+k_4)-k_{-2}k_3-2k_{-2}k_4}{k_2k_3+2k_2k_4} \quad \text{(eq. S73)}$$

$$\frac{\alpha_4}{\alpha_3}=\frac{k_1(k_3+k_4)-k_{-2}(k_3+2k_4)}{k_2(k_3+2k_4)} \quad \text{(eq. S74)}$$

$$\frac{\alpha_1\alpha_4}{\alpha_3}=\frac{k_2}{k_3+k_4}\left(\frac{k_1(k_3+k_4)-k_{-2}(k_3+2k_4)}{k_2(k_3+2k_4)}\right) \quad \text{(eq. S75)}$$

$$\frac{\alpha_1\alpha_4}{\alpha_3}=\frac{k_1(k_3+k_4)-k_{-2}(k_3+2k_4)}{(k_3+k_4)(k_3+2k_4)} \quad \text{(eq. S76)}$$

$$\frac{\alpha_4}{\alpha_3}<0 \Rightarrow \alpha_4<0 \Rightarrow k_1(k_3+k_4)-k_{-2}k_3-2k_{-2}k_4<0 \quad \text{(eq. S77)}$$

$$k_1<\frac{k_{-2}(k_3+2k_4)}{k_3+k_4} \quad \text{(eq. S78)}$$

$$\alpha_{11}=k_7\alpha_5-k_6-k_4\alpha_2-k_4\frac{\alpha_1\alpha_4}{\alpha_3}\geq 0 \quad \text{(eq. S79)}$$

$$k_1\geq k_6+\frac{k_4k_{-2}}{k_3+k_4}+k_4\left[\frac{k_1(k_3+k_4)-k_{-2}(k_3+2k_4)}{(k_3+k_4)(k_3+2k_4)}\right] \quad \text{(eq. S80)}$$

$$k_1(k_3+k_4)\geq k_6(k_3+2k_4) \Rightarrow k_6\leq\frac{k_1(k_3+k_4)}{k_3+2k_4} \quad \text{(eq. S81)}$$

Knowing that $k_1<\frac{k_{-2}(k_3+2k_4)}{k_3+k_4}$, it comes $k_6\leq k_{-2}$

$$[Au-S]=[A]+[G]+[C]+[E] \quad \text{(eq. S82)}$$

$$[Au-S]=\left([A]_0+\frac{\alpha_4}{\alpha_3}\right)e^{-\alpha_3t}-\frac{\alpha_4}{\alpha_3}+\alpha_5-\alpha_5e^{-k_7t}+\alpha_{12}e^{-k_5t}+\alpha_9e^{-\alpha_3t}-\alpha_{10}e^{-k_7t}+\alpha_{11}+\alpha_6-\alpha_6e^{-k_8t}=\left([A]_0+\frac{\alpha_4}{\alpha_3}+\alpha_9\right)e^{-\alpha_3t}-(\alpha_5+\alpha_{10})e^{-k_7t}+\alpha_{12}e^{-k_5t}-\alpha_6e^{-k_8t}+\alpha_6+\alpha_{11}+\alpha_5-\frac{\alpha_4}{\alpha_3}=\alpha_{15}e^{-\alpha_3t}-\alpha_{16}e^{-k_7t}+\alpha_{12}e^{-k_5t}-\alpha_6e^{-k_8t}+\alpha_6+\alpha_{11}+\alpha_5-\frac{\alpha_4}{\alpha_3} \quad \text{(eq. S83)}$$

$\alpha_{15}=\left([A]_0+\frac{\alpha_4}{\alpha_3}+\alpha_9\right)e^{-\alpha_3t}$ (eq. S84) $\qquad \alpha_{16}=\alpha_5+\alpha_{10}$ (eq. S85)

$t\rightarrow\infty$, $[Au-S]_\infty=\alpha_{11}+\alpha_6+\alpha_5-\frac{\alpha_4}{\alpha_3}>0$ (eq. S86)

$$[Au-S]_\infty=\frac{k_1}{k_7}+\frac{k_6}{k_8}+\frac{k_1}{k_5}-\frac{k_6}{k_5}-\frac{k_4k_{-2}}{k_5(k_3+k_4)}-\frac{k_4k_1}{k_5(k_3+2k_4)}+\frac{k_4k_{-2}}{k_5(k_3+k_4)}-\frac{k_1(k_3+k_4)}{k_2(k_3+2k_4)}+\frac{k_{-2}}{k_2}=\frac{k_1}{k_5}\left[\frac{(k_5+k_7)(k_3+2k_4)-k_4k_2k_7-k_5k_7(k_3+k_4)}{k_2k_7(k_3+2k_4)}\right]>\frac{k_2k_6(k_8-k_5)-k_5k_{-2}k_8}{k_2k_5k_8} \quad \text{(eq. S87)}$$

$$k_1>\frac{[k_2k_6(k_8-k_5)-k_5k_{-2}k_8](k_3+2k_4)k_7}{(k_5+k_7)(k_3+2k_4)-k_4k_2k_7-k_5k_7(k_3+k_4)} \quad \text{(eq. S88)}$$

To sum up all calculations for our kinetic study kinetic model should be described by next equations:

$$[NO_2] = [A] = \left([A]_0 + \frac{\alpha_4}{\alpha_3}\right)e^{\alpha_3 t} - \frac{\alpha_4}{\alpha_3} \quad \text{(eq. S89)}$$

$$[SH] = [C] = \alpha_{12}e^{-k_5 t} + \alpha_9 e^{-\alpha_3 t} - \alpha_{10}e^{-k_7 t} + \alpha_{11} \quad \text{(eq. S90)}$$

$$[SO_2] = [E] = \frac{k_6}{k_8}(1 - e^{-k_8 t}) \quad \text{(eq. S91)}$$

$$[N = N] = [G] + [C] + [E] = \alpha_{12}e^{-k_5 t} + \alpha_9 e^{-\alpha_3 t} - \alpha_{13}e^{-k_7 t} - \alpha_6 e^{-k_8 t} + \alpha_{14} \quad \text{(eq. S92)}$$

$$[Au - S] = [A] + [G] + [C] + [E] = \alpha_{15}t + \alpha_6 e^{-k_8 t} + \alpha_{14}e^{-k_7 t} - \alpha_{13}\, e^{-\alpha_3 t} - \alpha_{12}e^{-k_5 t} + \alpha_{16} \quad \text{(eq. S93)}$$

Where α_i meaning presented in Table S5.

Table S5. Overview of α_i equations applied for kinetics mode

$\alpha_1 = \frac{k_2}{k_3+k_4}$(eq. S41)	$\alpha_2 = \frac{k_{-2}}{k_3+k_4}$(eq. S42)	$\alpha_3 = k_2 + \frac{k_4k_2}{k_3+k_4}$(eq. S73)	$\alpha_4 = k_1 - k_{-2} - \frac{k_4k_{-2}}{k_3+k_4}$(eq. S32)
$\alpha_5 = \frac{k_1}{k_7}$(eq. S49)	$\alpha_6 = \frac{k_6}{k_8}$(eq. S52)	$\alpha_7 = k_4\alpha_1\left([A]_0 + \frac{\alpha_4}{\alpha_3}\right)$(eq. S56)	$\alpha_8 = k_7\alpha_5 - k_4\frac{\alpha_1\alpha_4}{\alpha_3} - k_4\alpha_2 - k_6$(eq. S57)
$\alpha_9 = \frac{\alpha_7}{k_5-\alpha_3}$(eq. S59)	$\alpha_{10} = \frac{k_7\alpha_5}{k_5-k_7}$(eq. S60)	$\alpha_{11} = \frac{\alpha_8}{k_5} = \frac{k_7\alpha_5}{k_5} - \frac{k_6}{k_5} - \frac{k_4\alpha_2}{k_5} - \frac{k_4\alpha_1\alpha_4}{k_5\alpha_3}$(eq. S69)	$\alpha_{12} = \alpha_{10} - \alpha_9 - \alpha_{11}$(eq. S65)
$\alpha_{13} = \alpha_5 + \alpha_{10}$ (eq. S68)	$\alpha_{14} = \alpha_5 + \alpha_{11} + \alpha_6$ (eq. S70)	$\alpha_{15} = \left([A]_0 + \frac{\alpha_4}{\alpha_3} + \alpha_9\right)e^{-\alpha_3 t}$(eq. S84)	$\alpha_{16} = \alpha_5 + \alpha_{10}$(eq. S85)

Taking into account that for [SH], [N=N] and [Au-S] kinetic models include too much numbers of α_i and k_i, values generated by fitting software will be strongly questionable.

Supplementary Note 11 – Density functional theory (DFT) calculations

Periodic DFT calculations were performed using the QuantumWise Atomistix ToolKit (QuantumATK) software[29] with projector augmented wave (PAW) [30] pseudopotentials (the cut-off energy = 500 eV). London dispersion corrections were included through Grimme's DFT-D3BJ [31] method.

To study mechanistic aspects of plasmonic excitation of Au NPs-PNTP, we created a model (Figure S17a) of the Au (111) slab structure (6×6) with 12 molecules of NTP was placed with a molecular rotation angle of 33° within commonly accepted c(4×2) and (√3×√3)-R30° thiolate-Au (111) interface [32]. Firstly, we analyzed the local density of states (LDOS) of adsorbed PNTP molecules by DFT calculations. The projected density of states (PDOS) plots for C, N, O, and Au atoms are shown in Figure S17b. On the Au(111) surface, the main peaks correspond to the frontier states of PNTP, which correspond to the HOMO and LUMO after hybridization with Au NPs. The energies upon hybridization were quantified as a maximum of the edged lines relative to the zero level in DOS vs energy correlations. The intensive peak near 2 eV indicates that an excitation energy of 1.95 eV (633 nm) is sufficient to excite absorbed PNTP. This is aligned with the generally considered thiolate-Au bond of 200 kJ/mol or 2eV [32].

Furthermore, the transition contribution map (TCM) was built to visualize the transitions as a function of the energies of the corresponding occupied and unoccupied states at an excitation energy of 1.96 eV. TCM is a versatile tool for plasmonic systems, which makes it possible to observe in real-time the relative contributions of each electron-to-hole transition to a specific photoabsorption peak (Figure S17c). The prominent red region indicates a significant transition occurring at an energy slightly above 1.90 eV, which corresponds to the energy of the irradiating light. This suggests that the system has absorbed the light and facilitated an electronic transition close to the energy of the incident photons. The observed transition could be due to localized surface plasmon resonance effects that enhance the molecular orbitals' interaction with the light, leading to a charge transfer excitation or chemical interface damping [33].

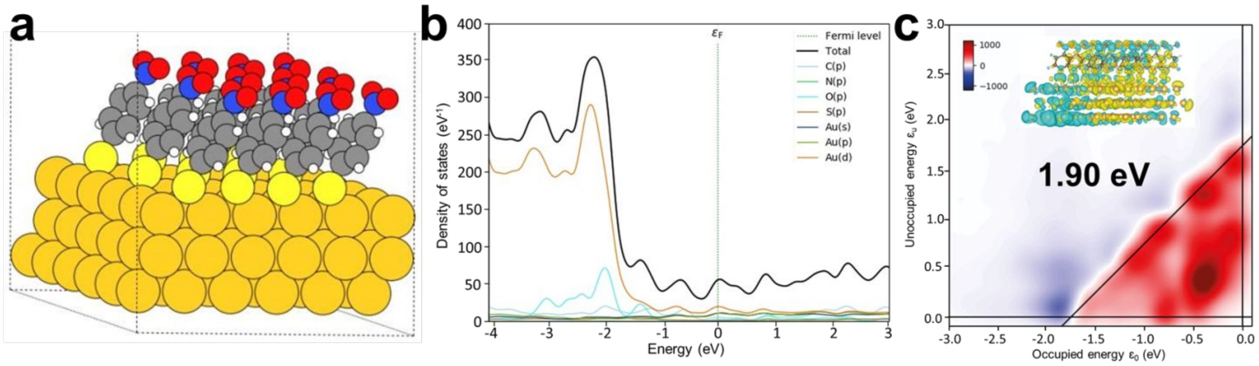

Figure S17. a) p-nitrothiophenol adsorbed on Au surface; b) Density of state curves; c) Transition contribution map

Supplementary Note 12 – Thermogravimetric analysis

A large batch (1000 mL) of Au NPs and Au-PNTP have been carefully washed to remove any residuals of stabilizing trisodium citrate (Figure S19a). Then, Au NPs and Au-NTP underwent heating with 10 °C/min from room to 800 °C in Ar. The comparison of thermograms revealed sharp weight loss in the region of 200-250 °C, where peak integration revealed T_{des}=223 °C (Figure S18). Exothermic process was detected at the region 200-250 °C at DSC curve of Au-NTP (Figure S19b). Therefore, in case of local heating over 223 °C, thiol desorption can occur.

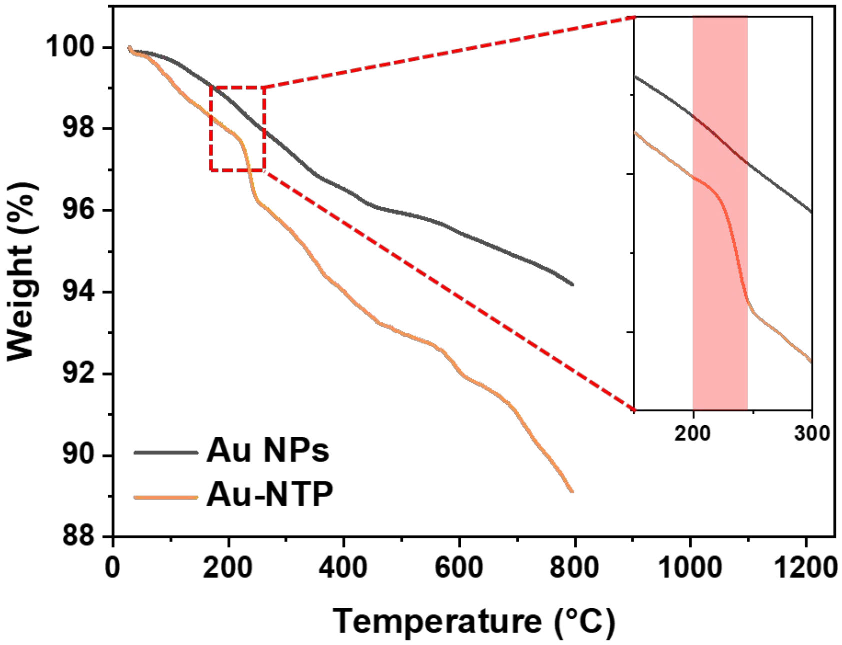

Figure S18. Thermograms of AuNPs and Au-NTP measured in air with 10 °C/min, where insert show the region of NTP desorption

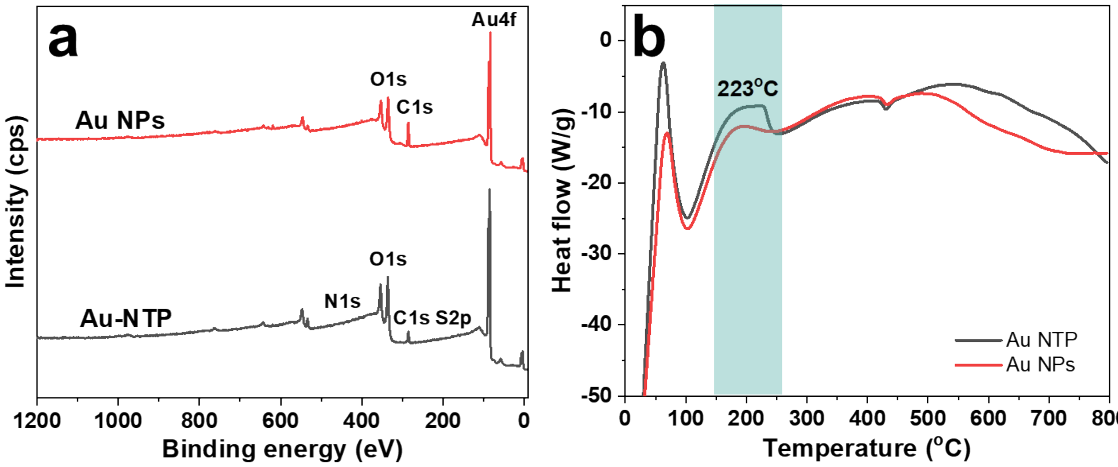

Figure S19. Characterization of Au-NTP: a) XPS survey spectra of AuNPs and AuNTP; b) DSC curves of Au NPs and AuNTP;

Supplementary Note 13 – Theoretical calculations of heating temperature

Theoretical temperature of heating were calculated starting with equation for max temperature in the center of solid heated by NPs [34]:

$$\Delta T^{top} \approx \frac{I_{inc}\rho_0}{2\kappa_h}[1 - e^{-H/\delta_{skin}}] \qquad \text{(eq. S94)}$$

where I_{inc} is illumination intensity of the laser; ρ_0 –radius of illuminated NPs; κ_h – thermal conductivity; δ_{skin} – skin depth experienced by the incident beam and H – thickness.

Taking into account that radius of illuminated NPs ρ_0 is equal 1.55 µm estimated by cross-sectional SEM on Figure S20) and δ_{skin} *is 3.4 nm* according to [35]:

$$\delta_{skin} = \sqrt{\frac{2\rho}{\omega\mu_0\mu_\tau}} \qquad \text{(eq.S95)}$$

where ρ, ω, μ_0, and μ_r are the conductor resistivity, current frequency, permeability of free space, and relative permeability of the conductor, respectively.

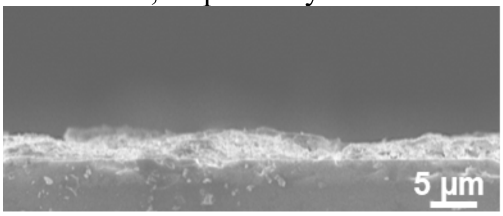

Figure S20. Cross-sectional SEM image of Au-NTP substrates

$H >> \delta_{skin}$ since we have multiple (potentially hundreds and thousands) layers of NPs, thus $e^{-H/\delta_{skin}} \rightarrow 0$. The NP layer behaves as porous medium with very high ratio of thermal conductance (305 W/m·K vs ≈0.024 W/m·K), which makes it very sensitive to geometry, etc. The effective thermal conductivity of saturated porous media continues to be one of the great unsolved problems in heat transfer science [34]. However, usually the heat conductivity of the medium is enhanced by a factor of 10-15 for the given ratio of conductivities, thus we can approximate $\kappa_h = 10\kappa_{air}$.

Therefore, final equation was obtained as:

$$\Delta T^{top} \approx \frac{I_{inc}\rho_0}{20\kappa_{air}} \qquad \text{(eq. S96)}$$

Table S6 show obtained local temperature depending on laser powers used in this research.

Table S6. Calculated local temperature depending of laser power.

**Power, kW/cm^2**	**ΔT^{top}, °C**
10	50
20	372
40	1019
80	2310

Supplementary Note 14 – Temperature-dependent Raman spectroscopy

The azo coupling reaction was performed under identical conditions (633 nm, 14 kW/cm², 60 s) at two different temperatures, 25 °C and 100 °C, represented in the Figure S21 by the orange and green lines/bars, respectively. Raman spectra were collected after the reaction using a laser power density of 3 kW/cm² to prevent additional DMAB formation. Following azo coupling at both temperatures, DMAB formation was observed, as indicated by the appearance of characteristic Raman peaks ν(C-N), β(CH) at 1135 cm^{-1} and ν(N=N) at 1391 and 1430 cm^{-1}.

To assess the temperature-dependent enhancement of the reaction, we compared the intensities of selected Raman peaks: Au-S at 330 cm^{-1}, C-S at 720 cm^{-1}, N=N at 1430 and NO_2 at 1330 cm^{-1}.

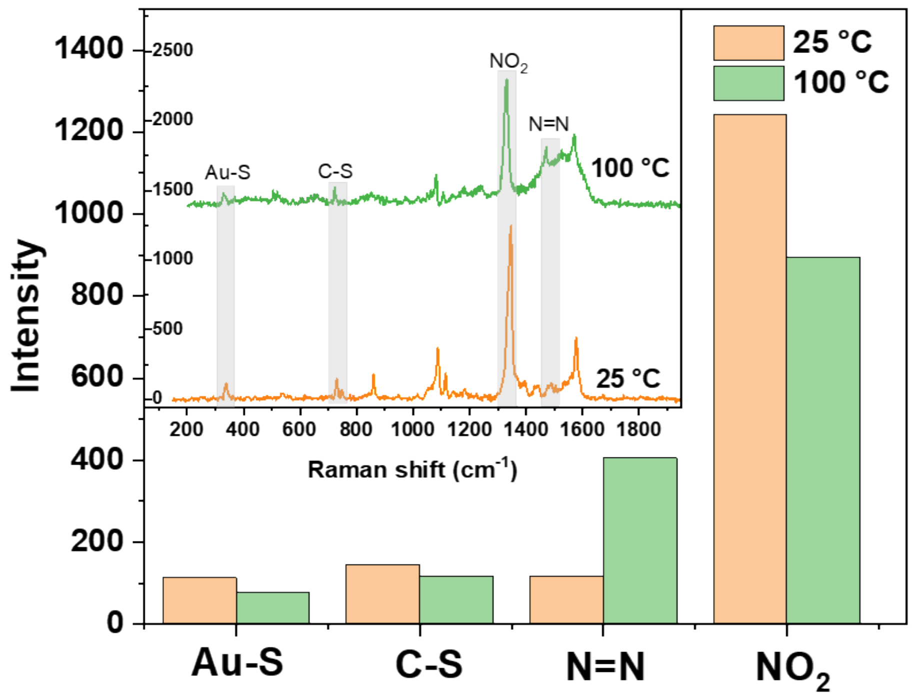

Figure S21. Raman spectra of Au-NTP after 60 seconds irradiation at power density14 kW/cm^2 under 25 and 100 °C.

Supplementary Note 15 – Plasmon-driven C-ON bond homolysis of alkoxyamine at different wavelength LED irradiation.

For kinetic measurements of alkoxyamine **SG1-St** homolysis under plasmonic excitation using AuNPs, the sampling was made during reaction time and probe was analyzed by EPR at 25 °C using SG1 (0.1 mM) as calibration standards. The detected concentration of released nitroxide SG1 allows plotting kinetic curves and calculate rate constants (Figure S22, Table S7) via exponential fitting and linearization as well.

In the control experiments in the absence of nanoparticles or in the dark, the nitroxide was not detected even in traces.

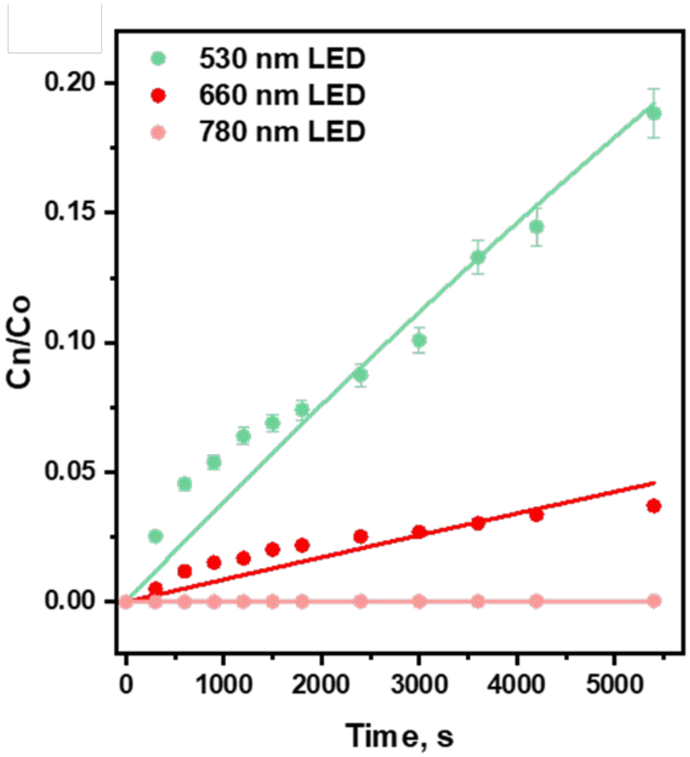

Figure S22. Kinetic curves of plasmon-induced homolysis of alkoxyamines at room temperature at different wavelength.

Supplementary Note 16 – Quantum Yield calculation

The quantum yields (QY) were calculated according to (eq.S97) (Table S7).

$$QY = \frac{C_{product} \cdot V \cdot N_a \cdot c \cdot h \cdot}{\Delta t \cdot P_{LED} \cdot \lambda} \cdot 100\% \qquad \text{(eq.S97)}$$

where C_{prod} – the nitroxide concentration; V – is the sample volume (L); N_a - the Avogadro constant; h – Planck's constant (J·s); c – the speed of light (m·s^{-1}); Δt – the illumination time (s); λ – the wavelength of the LED (m); and P_{LED} is the LED power (W·m^{-2}).

Table S7. Plasmon-driven homolysis at different wavelength of LEDs.

Wavelength, nm	k_d[a], s^{-1}	R^2	QY[c], %
530	4.4E-05	0.95	3.24E-04
660	8.7E-06	0.75	3.66E-05
780	6.4E-08[b]	-	2.96E-07

[a] Rate constants were obtained from exponential fitting of kinetic curves.

[b] As no homolysis was observed at 780 nm LED irradiation, the thermal value of k_d at 25 °C was used (E_a = 123kJ/mol)[36]

[c] QY were calculated with (eq. S97).

Supplementary Note 17 – Plasmon-driven C-ON bond homolysis of alkoxyamine at different power LED irradiation

For kinetic measurements, the sampling was made during reaction time and probe was analysed by EPR at 25°C using SG1 (0.1 mM) as calibration standards. The detected concentration of released nitroxide SG1 allows plotting kinetic curves and calculate rate constants (Figure S23, Table S8) via exponential fitting and linearization as well.

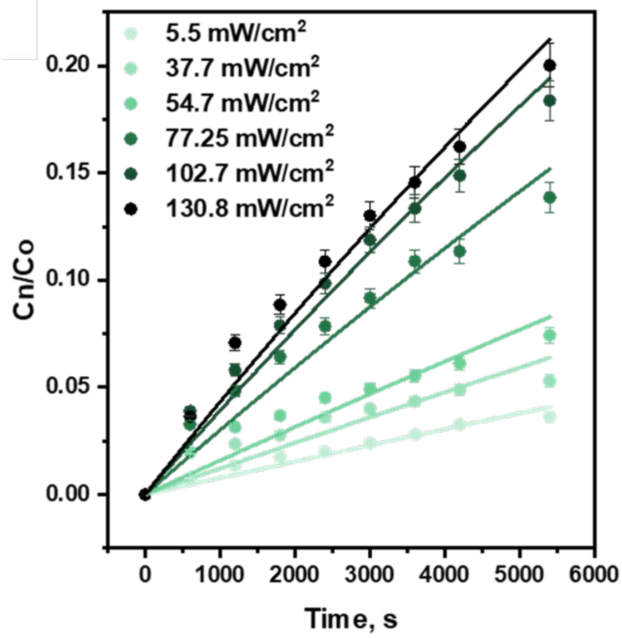

Figure S23. Kinetic curves of plasmon-induced homolysis of alkoxyamines at room temperature at different power of irradiation.

Table S8. Plasmon-driven homolysis at different power of 530 nm LED irradiation.

**Power, mW/cm²**	k_d, s^{-1}	R^2
5.5	7.7E-06	0.94
37.5	1.2E-05	0.80
54.7	1.6E-05	0.88
77.3	3.1E-05	0.95
102.7	4.0E-05	0.98
130.8	4.4E-05	0.97

To distinguish the nature of plasmon-induced process (photothermal or photochemical), a simple fitting tool was developed and is available at [37] based on previously reported procedure[38]. The app provides a user-friendly interface to the SciPy [39] curve fit function. It fits the Arrhenius model (eq. S98, with C, *Ea* and *A* as free parameters or fixed depending on user's consideration) and a simple linear model to the uploaded data, generating fit constants, plots, and R^2 values to assist in model selection.

$$k_d = A \cdot \exp\left(\frac{-Ea}{R \cdot (T_0 + C * I)}\right) \quad \text{(eq.S98)},$$

where k_d is the reaction rate constant of plasmon-induced homolysis, s^{-1}; A is the frequency factor for alkoxyamine C–ON bond homolysis (it was chosen to be fixed in our case for AAs homolysis at 2.4E14 s^{-1} [40,41]); E_a is the activation energy of the reaction; R is the ideal gas constant, (8.314 J · $K^{-1} \cdot mol^{-1}$); T is the temperature of plasmon-induced homolysis, (298 K); C is the heating coefficient. To perform the fitting, we fixed A, and E_a and C were kept as free parameters. Using our app, the E_a = 110.6 kJ/mol and C=0.0858 $K \cdot cm^2 \cdot mW^{-1}$ were calculated.

The optimal Arrhenius fitting solution was found when frequency factor was fixed at A=2.4E14 s^{-1} that represent the physical meaning. During fitting the C was fitted to be 0.0858 (that correspond to 13K heat over the rection) and E_a = 111 kJ/mol, which has a realistic value compared to previous reports, e.g., 123 kJ/mol in [36]. The obtained parameters seem to be in a good agreement with our experimental results.

References

(1) Turkevich, J.; Stevenson, P. C.; Hillier, J. A Study of the Nucleation and Growth Processes in the Synthesis of Colloidal Gold. *Discuss. Faraday Soc.* **1951**, *11*, 55. https://doi.org/10.1039/df9511100055.

(2) Matyjaszewski, K.; Woodworth, B. E.; Zhang, X.; Gaynor, S. G.; Metzner, Z. Simple and Efficient Synthesis of Various Alkoxyamines for Stable Free Radical Polymerization. *Macromolecules* **1998**, *31* (17), 5955–5957. https://doi.org/10.1021/ma9807264.

(3) Pavia, D. L.; Lampman, G. M.; Kriz, G. S. *Introduction to Spectroscopy: A Guide for Students of Organic Chemistry*, 3rd ed.; Thomson Learning: Washington, 2001.

(4) Thomas, M.; Mühlig, S.; Deckert-Gaudig, T.; Rockstuhl, C.; Deckert, V.; Marquetand, P. Distinguishing Chemical and Electromagnetic Enhancement in Surface-enhanced Raman Spectra: The Case of Para -nitrothiophenol. *J. Raman Spectrosc.* **2013**, *44* (11), 1497–1505. https://doi.org/10.1002/jrs.4377.

(5) Skadtchenko, B. O.; Aroca, R. Surface-Enhanced Raman Scattering of p-Nitrothiophenol. *Spectrochim. Acta Part A Mol. Biomol. Spectrosc.* **2001**, *57* (5), 1009–1016. https://doi.org/10.1016/S1386-1425(00)00415-7.

(6) Small, L. J.; Wheeler, D. R.; Spoerke, E. D. Nanoporous Membranes with Electrochemically Switchable, Chemically Stabilized Ionic Selectivity. *Nanoscale* **2015**, *7* (40), 16909–16920. https://doi.org/10.1039/C5NR02939B.

(7) Adenier, A.; Cabet-Deliry, E.; Chaussé, A.; Griveau, S.; Mercier, F.; Pinson, J.; Vautrin-Ul, C. Grafting of Nitrophenyl Groups on Carbon and Metallic Surfaces without Electrochemical Induction. *Chem. Mater.* **2005**, *17* (3), 491–501. https://doi.org/10.1021/cm0490625.

(8) Médard, C.; Morin, M. Chemisorption of Aromatic Thiols onto a Glassy Carbon Surface. *J. Electroanal. Chem.* **2009**, *632* (1–2), 120–126. https://doi.org/10.1016/j.jelechem.2009.04.005.

(9) Mohtasebi, A.; Chowdhury, T.; Hsu, L. H. H.; Biesinger, M. C.; Kruse, P. Interfacial Charge Transfer between Phenyl-Capped Aniline Tetramer Films and Iron Oxide Surfaces. *J. Phys. Chem. C* **2016**, *120* (51), 29248–29263. https://doi.org/10.1021/acs.jpcc.6b09950.

(10) Castner, D. G.; Hinds, K.; Grainger, D. W. X-Ray Photoelectron Spectroscopy Sulfur 2p Study of Organic Thiol and Disulfide Binding Interactions with Gold Surfaces. *Langmuir* **1996**, *12* (21), 5083–5086. https://doi.org/10.1021/la960465w.

(11) Munir, A.; Haq, T. ul; Qurashi, A.; Rehman, H. ur; Ul-Hamid, A.; Hussain, I. Ultrasmall Ni/NiO Nanoclusters on Thiol-Functionalized and -Exfoliated Graphene Oxide Nanosheets for Durable Oxygen Evolution Reaction. *ACS Appl. Energy Mater.* **2019**, *2* (1), 363–371. https://doi.org/10.1021/acsaem.8b01375.

(12) Wu, T.; Fitchett, C. M.; Downard, A. J. Para -Fluoro-Thiol Reaction on Anchor Layers Grafted from an Aryldiazonium Salt: A Tool for Surface Functionalization with Thiols. *Langmuir* **2021**, *37* (38), 11397–11405. https://doi.org/10.1021/acs.langmuir.1c02012.

(13) Liu, G.; Liu, J.; Böcking, T.; Eggers, P. K.; Gooding, J. J. The Modification of Glassy Carbon and Gold Electrodes with Aryl Diazonium Salt: The Impact of the Electrode Materials on the Rate of Heterogeneous Electron Transfer. *Chem. Phys.* **2005**, *319* (1–3), 136–146. https://doi.org/10.1016/j.chemphys.2005.03.033.

(14) Uzun, D.; Arslan, H.; Gündüzalp, A. B.; Hasdemir, E. Preparation of Modified Glassy Carbon Surface with N-(1-H-Indole-3yl) Methylene Thiazole-2-Amine and Its Characterization. *Surf. Coatings Technol.* **2014**, *239*, 108–115. https://doi.org/10.1016/j.surfcoat.2013.11.028.

(15) Liu, Y.-C.; McCreery, R. L. Reactions of Organic Monolayers on Carbon Surfaces Observed with Unenhanced Raman Spectroscopy. *J. Am. Chem. Soc.* **1995**, *117* (45), 11254–11259. https://doi.org/10.1021/ja00150a024.

(16) Schumacher, L.; Jose, J.; Janoschka, D.; Dreher, P.; Davis, T. J.; Ligges, M.; Li, R.; Mo, M.; Park, S.; Shen, X.; Weathersby, S.; Yang, J.; Wang, X.; Meyer zu Heringdorf, F.;

Sokolowski-Tinten, K.; Schlücker, S. Precision Plasmonics with Monomers and Dimers of Spherical Gold Nanoparticles: Nonequilibrium Dynamics at the Time and Space Limits. *J. Phys. Chem. C* **2019**, *123* (21), 13181–13191. https://doi.org/10.1021/acs.jpcc.9b01007.

(17) Erzina, M.; Guselnikova, O.; Postnikov, P.; Elashnikov, R.; Kolska, Z.; Miliutina, E.; Švorčík, V.; Lyutakov, O. Plasmon-Polariton Induced, "from Surface" RAFT Polymerization, as a Way toward Creation of Grafted Polymer Films with Thickness Precisely Controlled by Self-Limiting Mechanism. *Adv. Mater. Interfaces* **2018**, *5* (22). https://doi.org/10.1002/admi.201801042.

(18) Zhou, B.; Ou, W.; Shen, J.; Zhao, C.; Zhong, J.; Du, P.; Bian, H.; Li, P.; Yang, L.; Lu, J.; Li, Y. Y. Controlling Plasmon-Aided Reduction of p-Nitrothiophenol by Tuning the Illumination Wavelength. *ACS Catal.* **2021**, *11* (24), 14898–14905. https://doi.org/10.1021/acscatal.1c04091.

(19) Koopman, W.; Titov, E.; Sarhan, R. M.; Gaebel, T.; Schürmann, R.; Mostafa, A.; Kogikoski, S.; Milosavljević, A. R.; Stete, F.; Liebig, F.; Schmitt, C. N. Z.; Koetz, J.; Bald, I.; Saalfrank, P.; Bargheer, M. The Role of Structural Flexibility in Plasmon-Driven Coupling Reactions: Kinetic Limitations in the Dimerization of Nitro-Benzenes. *Adv. Mater. Interfaces* **2021**, *8* (22), 2101344. https://doi.org/10.1002/admi.202101344.

(20) Zhang, Q.; Blom, D. A.; Wang, H. Nanoporosity-Enhanced Catalysis on Subwavelength Au Nanoparticles: A Plasmon-Enhanced Spectroscopic Study. *Chem. Mater.* **2014**, *26* (17), 5131–5142. https://doi.org/10.1021/cm502508d.

(21) Wang, J. L.; Ando, R. A.; Camargo, P. H. C. Investigating the Plasmon-Mediated Catalytic Activity of AgAu Nanoparticles as a Function of Composition: Are Two Metals Better than One? *ACS Catal.* **2014**, *4* (11), 3815–3819. https://doi.org/10.1021/cs501189m.

(22) Schürmann, R.; Ebel, K.; Nicolas, C.; Milosavljević, A. R.; Bald, I. Role of Valence Band States and Plasmonic Enhancement in Electron-Transfer-Induced Transformation of Nitrothiophenol. *J. Phys. Chem. Lett.* **2019**, *10* (11), 3153–3158. https://doi.org/10.1021/acs.jpclett.9b00848.

(23) Choi, H. K.; Lee, K. S.; Shin, H. H.; Kim, Z. H. Identification of the First Elementary Step in the Photocatalytic Reduction of Nitrobenzenethiols on a Metallic Surface. *J. Phys. Chem. Lett.* **2016**, *7* (20), 4099–4104. https://doi.org/10.1021/acs.jpclett.6b01852.

(24) Choi, H.-K.; Park, W.-H.; Park, C.-G.; Shin, H.-H.; Lee, K. S.; Kim, Z. H. Metal-Catalyzed Chemical Reaction of Single Molecules Directly Probed by Vibrational Spectroscopy. *J. Am. Chem. Soc.* **2016**, *138* (13), 4673–4684. https://doi.org/10.1021/jacs.6b01865.

(25) Zhang, Q.; Wang, H. Mechanistic Insights on Plasmon-Driven Photocatalytic Oxidative Coupling of Thiophenol Derivatives: Evidence for Steady-State Photoactivated Oxygen. *J. Phys. Chem. C* **2018**, *122* (10), 5686–5697. https://doi.org/10.1021/acs.jpcc.8b00660.

(26) Carlini, L.; Fasolato, C.; Postorino, P.; Fratoddi, I.; Venditti, I.; Testa, G.; Battocchio, C. Comparison between Silver and Gold Nanoparticles Stabilized with Negatively Charged Hydrophilic Thiols: SR-XPS and SERS as Probes for Structural Differences and Similarities. *Colloids Surfaces A Physicochem. Eng. Asp.* **2017**, *532*, 183–188. https://doi.org/10.1016/j.colsurfa.2017.05.045.

(27) Varnholt, B.; Oulevey, P.; Luber, S.; Kumara, C.; Dass, A.; Bürgi, T. Structural Information on the Au–S Interface of Thiolate-Protected Gold Clusters: A Raman Spectroscopy Study. *J. Phys. Chem. C* **2014**, *118* (18), 9604–9611. https://doi.org/10.1021/jp502453q.

(28) George Socrates. *Infrared and Raman Characteristic Group Frequencies: Tables and Charts*; Wiley-VCH Verlag, 2004.

(29) Smidstrup, S.; Markussen, T.; Vancraeyveld, P.; Wellendorff, J.; Schneider, J.; Gunst, T.; Verstichel, B.; Stradi, D.; Khomyakov, P. A.; Vej-Hansen, U. G.; Lee, M.-E.; Chill, S. T.; Rasmussen, F.; Penazzi, G.; Corsetti, F.; Ojanperä, A.; Jensen, K.; Palsgaard, M. L. N.; Martinez, U.; Blom, A.; Brandbyge, M.; Stokbro, K. QuantumATK: An Integrated Platform of Electronic and Atomic-Scale Modelling Tools. *J. Phys. Condens. Matter* **2020**, *32* (1), 015901. https://doi.org/10.1088/1361-648X/ab4007.

(30) Blöchl, P. E. Projector Augmented-Wave Method. *Phys. Rev. B* **1994**, *50* (24), 17953–17979. https://doi.org/10.1103/PhysRevB.50.17953.
(31) Grimme, S.; Ehrlich, S.; Goerigk, L. Effect of the Damping Function in Dispersion Corrected Density Functional Theory. *J. Comput. Chem.* **2011**, *32* (7), 1456–1465. https://doi.org/10.1002/jcc.21759.
(32) Pensa, E.; Cortés, E.; Corthey, G.; Carro, P.; Vericat, C.; Fonticelli, M. H.; Benítez, G.; Rubert, A. A.; Salvarezza, R. C. The Chemistry of the Sulfur–Gold Interface: In Search of a Unified Model. *Acc. Chem. Res.* **2012**, *45* (8), 1183–1192. https://doi.org/10.1021/ar200260p.
(33) Fusco, Z.; Catchpole, K.; Beck, F. J. Investigation of the Mechanisms of Plasmon-Mediated Photocatalysis: Synergistic Contribution of near-Field and Charge Transfer Effects. *J. Mater. Chem. C* **2022**, *10* (19), 7511–7524. https://doi.org/10.1039/D2TC00491G.
(34) Un, I. W.; Sivan, Y. Parametric Study of Temperature Distribution in Plasmon-Assisted Photocatalysis. *Nanoscale* **2020**, *12* (34), 17821–17832. https://doi.org/10.1039/D0NR03897K.
(35) Hajar, Z.; Majid, V. Self-Magnetism of Skin Effect as a Function of Nanoparticle Diameter on Absorption Frequency. *Plasmonics* **2017**, *12* (5), 1523–1528. https://doi.org/10.1007/s11468-016-0414-z.
(36) Audran, G.; Brémond, P.; Joly, J.-P.; Marque, S. R. A.; Yamasaki, T. C–ON Bond Homolysis in Alkoxyamines. Part 12: The Effect of the Para-Substituent in the 1-Phenylethyl Fragment. *Org. Biomol. Chem.* **2016**, *14* (14), 3574–3583. https://doi.org/10.1039/C6OB00384B.
(37) *Plasmon Catalysis Data Fitting*. https://curvefitapp-plasmon.streamlit.app (accessed 2025-03-26).
(38) Christopher, P.; Xin, H.; Linic, S. Visible-Light-Enhanced Catalytic Oxidation Reactions on Plasmonic Silver Nanostructures. *Nat. Chem.* **2011**, *3* (6), 467–472. https://doi.org/10.1038/nchem.1032.
(39) Virtanen, P.; Gommers, R.; Oliphant, T. E.; Haberland, M.; Reddy, T.; Cournapeau, D.; Burovski, E.; Peterson, P.; Weckesser, W.; Bright, J.; van der Walt, S. J.; Brett, M.; Wilson, J.; Millman, K. J.; Mayorov, N.; Nelson, A. R. J.; Jones, E.; Kern, R.; Larson, E.; Carey, C. J.; Polat, İ.; Feng, Y.; Moore, E. W.; VanderPlas, J.; Laxalde, D.; Perktold, J.; Cimrman, R.; Henriksen, I.; Quintero, E. A.; Harris, C. R.; Archibald, A. M.; Ribeiro, A. H.; Pedregosa, F.; van Mulbregt, P.; Vijaykumar, A.; Bardelli, A. Pietro; Rothberg, A.; Hilboll, A.; Kloeckner, A.; Scopatz, A.; Lee, A.; Rokem, A.; Woods, C. N.; Fulton, C.; Masson, C.; Häggström, C.; Fitzgerald, C.; Nicholson, D. A.; Hagen, D. R.; Pasechnik, D. V.; Olivetti, E.; Martin, E.; Wieser, E.; Silva, F.; Lenders, F.; Wilhelm, F.; Young, G.; Price, G. A.; Ingold, G.-L.; Allen, G. E.; Lee, G. R.; Audren, H.; Probst, I.; Dietrich, J. P.; Silterra, J.; Webber, J. T.; Slavič, J.; Nothman, J.; Buchner, J.; Kulick, J.; Schönberger, J. L.; de Miranda Cardoso, J. V.; Reimer, J.; Harrington, J.; Rodríguez, J. L. C.; Nunez-Iglesias, J.; Kuczynski, J.; Tritz, K.; Thoma, M.; Newville, M.; Kümmerer, M.; Bolingbroke, M.; Tartre, M.; Pak, M.; Smith, N. J.; Nowaczyk, N.; Shebanov, N.; Pavlyk, O.; Brodtkorb, P. A.; Lee, P.; McGibbon, R. T.; Feldbauer, R.; Lewis, S.; Tygier, S.; Sievert, S.; Vigna, S.; Peterson, S.; More, S.; Pudlik, T.; Oshima, T.; Pingel, T. J.; Robitaille, T. P.; Spura, T.; Jones, T. R.; Cera, T.; Leslie, T.; Zito, T.; Krauss, T.; Upadhyay, U.; Halchenko, Y. O.; Vázquez-Baeza, Y. SciPy 1.0: Fundamental Algorithms for Scientific Computing in Python. *Nat. Methods* **2020**, *17* (3), 261–272. https://doi.org/10.1038/s41592-019-0686-2.
(40) *Encyclopedia of Radicals in Chemistry, Biology and Materials*; Chatgilialoglu, C., Studer, A., Eds.; Wiley, 2012. https://doi.org/10.1002/9781119953678.
(41) Bertin, D.; Gigmes, D.; Marque, S. R. A.; Tordo, P. Polar, Steric, and Stabilization Effects in Alkoxyamines C−ON Bond Homolysis: A Multiparameter Analysis. *Macromolecules* **2005**, *38* (7), 2638–2650. https://doi.org/10.1021/ma050004u.
